# Advancements in nanoparticle-based treatment approaches for skin cancer therapy

**DOI:** 10.1186/s12943-022-01708-4

**Published:** 2023-01-12

**Authors:** Leli Zeng, B. H. Jaswanth Gowda, Mohammed Gulzar Ahmed, Mohammed A. S. Abourehab, Zhe-Sheng Chen, Changhua Zhang, Jia Li, Prashant Kesharwani

**Affiliations:** 1 Guangdong Provincial Key Laboratory of Digestive Cancer Research, Digestive Diseases Center, The Seventh Affiliated Hospital of Sun Yat-Sen University, Shenzhen, Guangdong 518107 China; 2 Department of Pharmaceutics, Yenepoya Pharmacy College & Research Centre, Yenepoya (Deemed to Be University), Mangalore, 575018 Karnataka India; 3 Department of Pharmaceutics, College of Pharmacy, Umm Al-Qura University, Makkah, 21955 Saudi Arabia; 4 Department of Pharmaceutical Sciences, College of Pharmacy and Health Sciences, Jamaica, NY 11439 USA; 5 Department of Pharmaceutics, School of Pharmaceutical Education and Research, Jamia Hamdard, New Delhi, 110062 India; 6 Department of Pharmacology, Center for Transdisciplinary Research, Saveetha Dental College, Saveetha Institute of Medical and Technical Science, Chennai, India

**Keywords:** Nanotechnology, Nanomaterials, Metal nanoparticles, Skin carcinoma, Melanoma, Polymer

## Abstract

Skin cancer has emerged as the fifth most commonly reported cancer in the world, causing a burden on global health and the economy. The enormously rising environmental changes, industrialization, and genetic modification have further exacerbated skin cancer statistics. Current treatment modalities such as surgery, radiotherapy, conventional chemotherapy, targeted therapy, and immunotherapy are facing several issues related to cost, toxicity, and bioavailability thereby leading to declined anti-skin cancer therapeutic efficacy and poor patient compliance. In the context of overcoming this limitation, several nanotechnological advancements have been witnessed so far. Among various nanomaterials, nanoparticles have endowed exorbitant advantages by acting as both therapeutic agents and drug carriers for the remarkable treatment of skin cancer. The small size and large surface area to volume ratio of nanoparticles escalate the skin tumor uptake through their leaky vasculature resulting in enhanced therapeutic efficacy. In this context, the present review provides up to date information about different types and pathology of skin cancer, followed by their current treatment modalities and associated drawbacks. Furthermore, it meticulously discusses the role of numerous inorganic, polymer, and lipid-based nanoparticles in skin cancer therapy with subsequent descriptions of their patents and clinical trials.

**Graphical Abstract:**

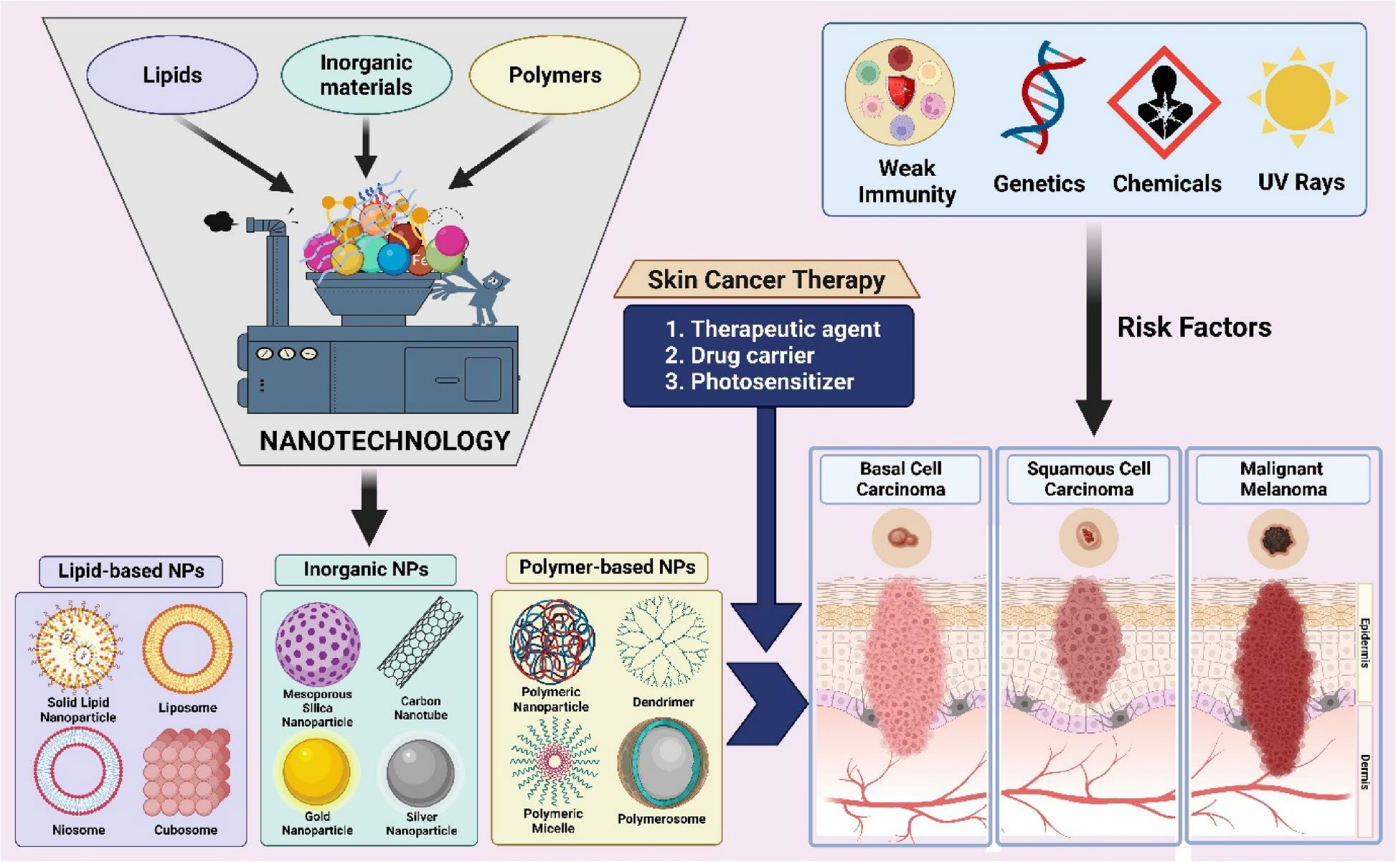

## Introduction

Skin carcinoma is one of the most dangerous types of cancer that was described by Laennec (melanoma), Jacob (basal cell carcinoma), and Bowen (squamous cell carcinoma in situ) in the years 1804, 1827, and 1912, respectively [1–4]. As of 2020, skin carcinoma is the fifth most commonly reported cancer in the world, according to World Health Organization [5]. In 2022, the American Academy of Dermatology (AAD) disclosed that approximately 9,500 people in the United States are diagnosed with skin cancer every day. AAD also stated that at least one in five Americans would develop skin cancer in their lifetime [6, 7]. Other than the United States, the highest incidence rate of skin cancer is also perceived in Australia and New Zealand, with an average case of 33 per 1,00,000 residents, followed by countries like Norway and Denmark (northern European countries) [5, 8]. Some of the proven risk factors for skin cancer include exposure to ultraviolet radiation [9, 10], chemical carcinogens [11, 12], genetic modulation [13, 14], fair skin [15], immunosuppression [16–18], etc. Based on the cellular origin, skin cancer is categorized into two types, i.e., melanoma skin cancer (melanocytes) and non-melanoma skin cancer (keratinocytes). Further, based on severity, non-melanoma skin cancer is divided into basal cell carcinoma (BCC) and squamous cell carcinoma (SCC) [19]. Although non-melanoma skin cancer accounts for 95% (BCC: 75%, SCC: 20%) of all reported skin cancer cases, the vast majority of skin cancer deaths are due to melanoma (80% death rate), which is a serious medical issue [20].

Currently, the most commonly employed treatment strategies for skin cancer during its initial stages include excision surgery [21], Mohs surgery [22], radiation therapy [23], curettage and electrodesiccation [24], cryotherapy [25], and photodynamic therapy [26]. However, in advanced stages where surgery and radiotherapy are impossible, immunotherapy (I) [27], targeted therapy (T) [28], and chemotherapy (C) [29] are widely utilized. Nonetheless, even after surgery and radiotherapy, the ITC is preferred chiefly to abolish the recurrence of skin cancer sooner or later. But the drawbacks associated with immunotherapy and targeted therapy, such as poor bioavailability and high cost, turn the patient’s eyes towards chemotherapy [30–32]. Although chemotherapy dramatically reduces the treatment cost of skin cancer, it suffers from poor therapeutic efficacy followed by causing severe side effects due to tumor resistance, inadequate solubility and permeability, poor bioavailability, non-targetability, and so on [33]. Lastly, the American Cancer Society states that the five-year survival rate for melanoma that spreads to regional and distant lymph nodes (advanced stage) is 68% and 30%, respectively, with the current treatment strategies [34]. Thus, an immediate call needs to be made to devise a groundbreaking treatment approach to diminish skin cancer conditions regardless of their advanced stages.

Nanotechnology has gained significant attention in various biomedical applications, including cancer therapy, due to its ability to deal with materials in size range of 1–1000 nm [35–37]. The nano-sized materials possess unique physicochemical properties that can immensely improvise the efficacy of cancer therapeutics. Many nanomaterials such as nanofibers [38], nanosuspension [39], nanoemulsions [40], and nanoclay [41] have been widely exploited for the treatment of skin cancer. However, nanoparticles (NPs) have shown exceptional supremacy over all other nanomaterials [42]. Further, the ability of NPs to act as an anticancer agent (due to their intrinsic therapeutic property), encapsulate and safeguard therapeutic moieties (hydrophilic and lipophilic), target the tumor (via active or passive approach), overcome the chemoresistance (to enhance the tumor cell uptake), control the drug release, and increase the skin permeability (to improve the topical/transdermal delivery of anticancer agents) has made them predominant candidates in skin cancer therapy [39, 43].

Nevertheless, nanotechnology in cancer therapy is not a modest approach, and already there are few NPs such as Doxil® (PEGylated liposome loaded with doxorubicin – 1995), Abraxane® (albumin-bound NPs loaded with paclitaxel – 2005), Oncaspar® (polymer protein conjugated with L-asparaginase – 2006), Marqibo® (liposome loaded with vincristine – 2012), Onivyde® (liposome loaded with Irinotecan – 2015), and Vyxeos® (liposome loaded with Cytarabine/ Daunorubicin – 2017) that were approved by Food and Drug Administration (FDA). Additionally, NanoTherm® (iron oxide NPs – 2010) and Hensify® (hafinum oxide NPs – 2019) are some of the inorganic NPs that were approved by European Medicines Agency (EMA). However, they are specifically intended for use in breast cancer, ovarian cancer, non-small-cell lung carcinoma, sarcoma, glioblastoma, pancreatic cancer, leukemia, multiple myeloma, and so on, but not for skin cancer [44–46]. Thus, many researchers and pharmaceutical companies are still striving to come up with NP-based treatment modality for efficient treatment of skin cancer by overcoming the toxicity barrier. With this contemplate, the present review provides a brief insight into various skin cancer types and pathology. Further, the authors have summarized the current treatment strategies for skin cancer along with their drawbacks. In later sections, the ambit of nanotechnology and various categories of NPs in skin cancer therapy are rigorously canvassed based on the most recent literatures followed by a detailed description of recent patents and clinical trials. Although several reviews have already elaborated on the role of nanotechnology in skin cancer, the originality of the present review lies in the detailed classification of NPs, such as inorganic, polymer, and lipid-based NPs, which makes it a state-of-the-art review.

## Types and pathology of skin cancer

### Basal cell carcinoma

**Fig. 1:**
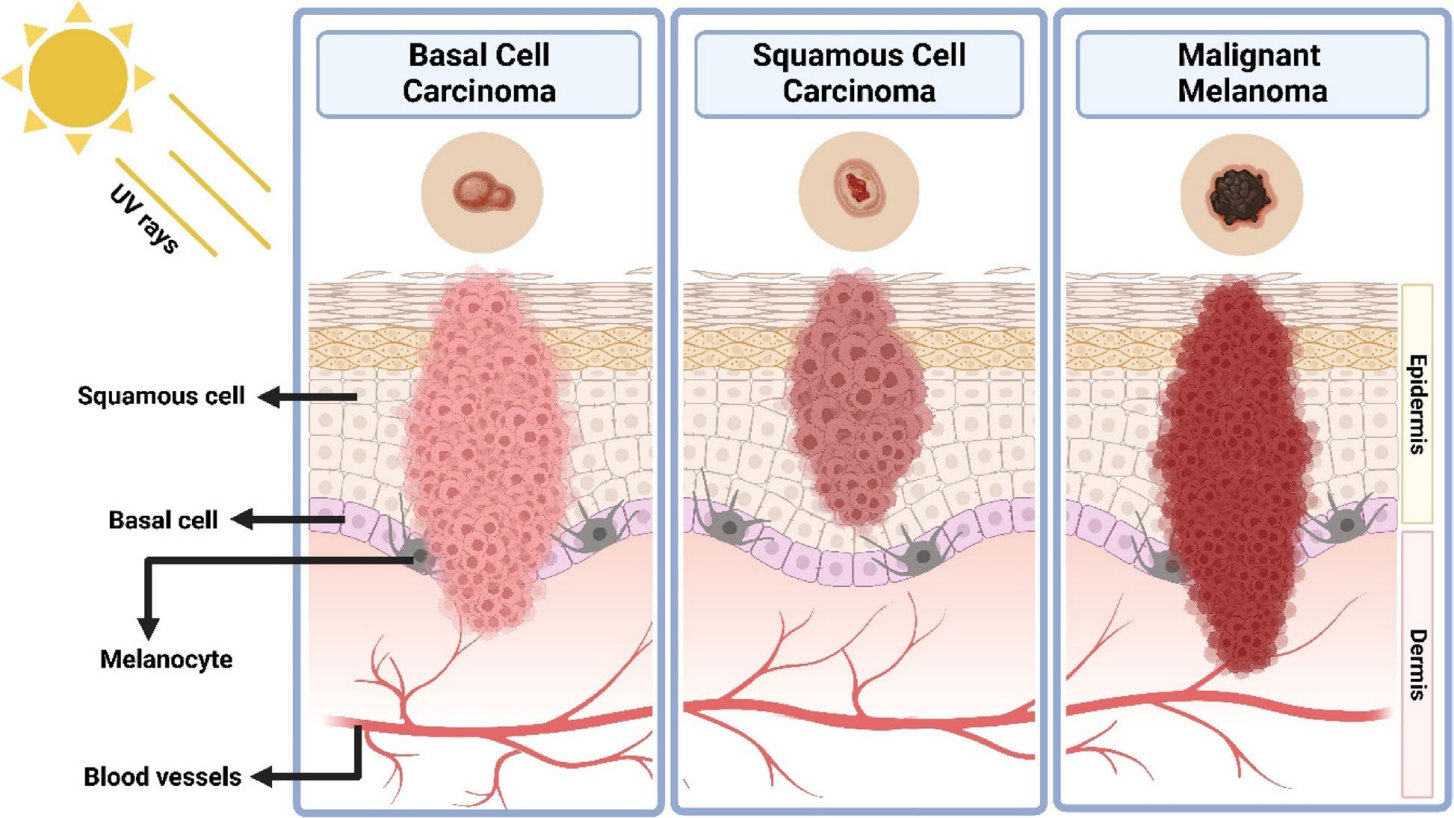
Diagrammatic representation of basal cell carcinoma, squamous cell carcinoma, and melanoma

Basal cell carcinoma (BCC) is the commonest (accounts for 70% of cutaneous malignancies) and least aggressive skin tumor that predominantly occurs in the region subject to extreme sun exposure, specifically on the neck and head [47]. Since this carcinoma arises from the basal layer of cells in epidermis, it has been termed basal cell carcinoma. The mutation and inactivation of p53 tumor suppressor gene, Ras protein, and sonic hedgehog glycoprotein caused by ultraviolet B radiation are estimated to be the mechanism behind the development of BCC. Additionally, its genesis is linked with germ cells of the hair follicle [48]. Based on their morphology, risk of recurrence, and metastasis, they have been categorized into several subtypes, such as nodular, superficial, micronodular, and infiltrative BCC. The nodular BCC tends to recur less frequently compared to other subtypes since they are clinically known, and the lesion boundary is well defined for precise treatment. The superficial BCC is characterized by a smooth or red stain in the epidermis with limited or nil invasion into the dermis (Fig. 1). Unlike other subtypes, which are formed by large aggregates, the micronodular BCC is constituted by the aggregates of small and round basaloid cells. Lastly, as the name suggests, infiltrative BCC invades both peripheral and deep regions of the skin, even penetrating the dermis, making them the most aggressive subtype [49]. Some of the individual risk factors for BCC involves genetic condition like Gorlin-Goltz syndrome, age, gender, immunosuppression, ultraviolet radiation, Fitzpatrick skin types I and II, and so on [50].

### Squamous cell carcinoma

Squamous cell carcinoma (SCC) is the second most frequently occurring skin cancer (accounts for 25% of cutaneous malignancies) following BCC and is more highly invasive than BCC [51]. The cervicofacial regions such as ears and lower lip are highly susceptible to developing SCC than BCC. Unlike BCC, inactivation of E-cadherin protein along with mutation of p53 tumor suppressor gene, Ras protein plays a significant role in developing SCC. SCC is distinguished by an atypical proliferation of invasive squamous cells that could metastasize into various parts of the body (Fig. 1) [52]. The aggressiveness of SCC depends on the location, depth, size, and differentiation of lesion. For instance, lesions beyond 2 cm in diameter and 4 mm in depth have greater chances of recurrence and metastasis. With respect to differentiation, a fully-defined SCC has distinct cytology, irregular neoplastic keratinocyte infiltration of the dermis, and varying degrees of inflammation and fibrosis underneath the tumor. However, deeper invasion and increased mitotic activity, including blood vessel invasion, are characteristics of moderately-defined SCC. Nevertheless, the least-defined SCC commonly invades the hypodermis and has negligible keratinization. Similar to BCC, the main reason behind the occurrence of SCC is immoderate exposure to ultraviolet radiation. But, other factors such as human papillomavirus (HPV), chemical carcinogens, genodermatoses, inflammatory conditions, and medicaments (tumor necrosis factor—α inhibitors) also hold responsible for SCC [53, 54].

### Melanoma

**Fig. 2:**
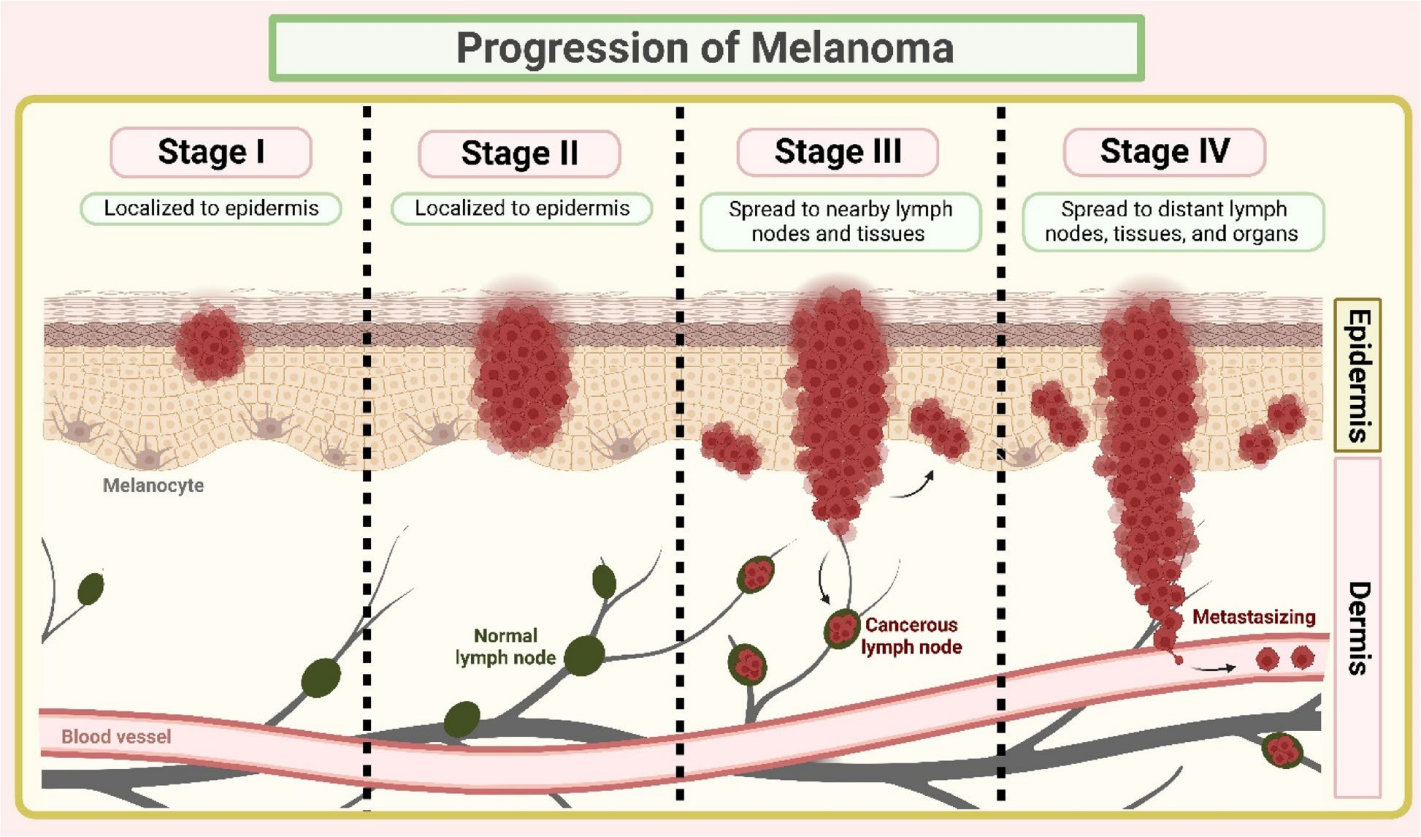
An illustration of melanoma progression

Melanoma is the least common type of skin cancer (5% of cutaneous malignancies), yet the most aggressive one, accounts for about 80% of overall skin cancer deaths [55, 56]. Melanoma arises from pigment (melanin) producing cells called melanocytes with uncontrollable division causing metastatic events (Fig. 1) [57]. During the initial stages, the lesion will be flat and pigmented with an indistinct shape and also limited to the epidermis. At later stages, the tumor growth will be vertical, infiltrating into the collagen fibers in the dermal layer. Lastly, the tumor infiltrates the subcutis to produce nodules and papules [56, 58]. The actual stages of melanoma are shown in Table 1 and Fig. 2.

**Table 1:** Stages of melanoma as per American Cancer Society

**Melanoma stage**	**Description**
0	Tumor invades the skin surface (epidermis) with slow mitotic rate. Not spread to nearby lymph or distant tissues/organs. This stage is also termed “melanoma in situ.”
I	Horizontal expansion of tumor on skin surface. Not more than 2 mm in thickness and might or might not be ulcerated. Not spread to nearby lymph or distant tissues/organs
II	Vertical expansion of tumor, thickness ranging from minimum 1 mm to more than 4 mm. Ulcerated or non-ulcerated. Not spread to nearby lymph or distant tissues/organs
III A	Tumor with not more than 2 mm thickness. Ulcerated or non-ulcerated. Cancer has spread to 1–3 nearby lymph nodes (can only be seen under microscope). Not spread to distant tissues/organs
III B	Tumor with not more than 4 mm thickness. Ulcerated or non-ulcerated. Cancer has spread to 1 nearby lymph node and small areas of nearby skin. Not spread to distant tissues/organs
III C	Tumor with not more than 4 mm thickness. Ulcerated or non-ulcerated. Cancer has spread to small areas of nearby skin and 4 or more nearby lymph nodes. Not spread to distant tissues/organs
III D	Tumor with more than 4 mm thickness and ulcerated. Cancer has spread to small areas of nearby skin and 4 or more nearby lymph nodes. Not spread to distant tissues/organs
IV	Tumor can have any thickness. Cancer has spread to nearby lymph nodes and distant organs such as brain, liver, lungs, bone, or kidney

In the United States, patients with advanced stages of melanoma have shown survival rates of 3 to 11 months. After diagnosis, the five-year survival rate of patients with metastatic melanoma was less than < 10%. The patients suffering from stage I and II melanoma displayed a five-year survival rate of 99.4%, followed by 68.0% and 29.4% for stage III and IV, respectively [6]. Some risk factors for melanoma include ultraviolet radiation, genetics, fair skin, chemical carcinogens, and immunosuppression. In addition, evidence supported that indoor tanning was also responsible for melanoma occurrence [59].

## Current treatment approaches and their limitations

**Fig. 3:**
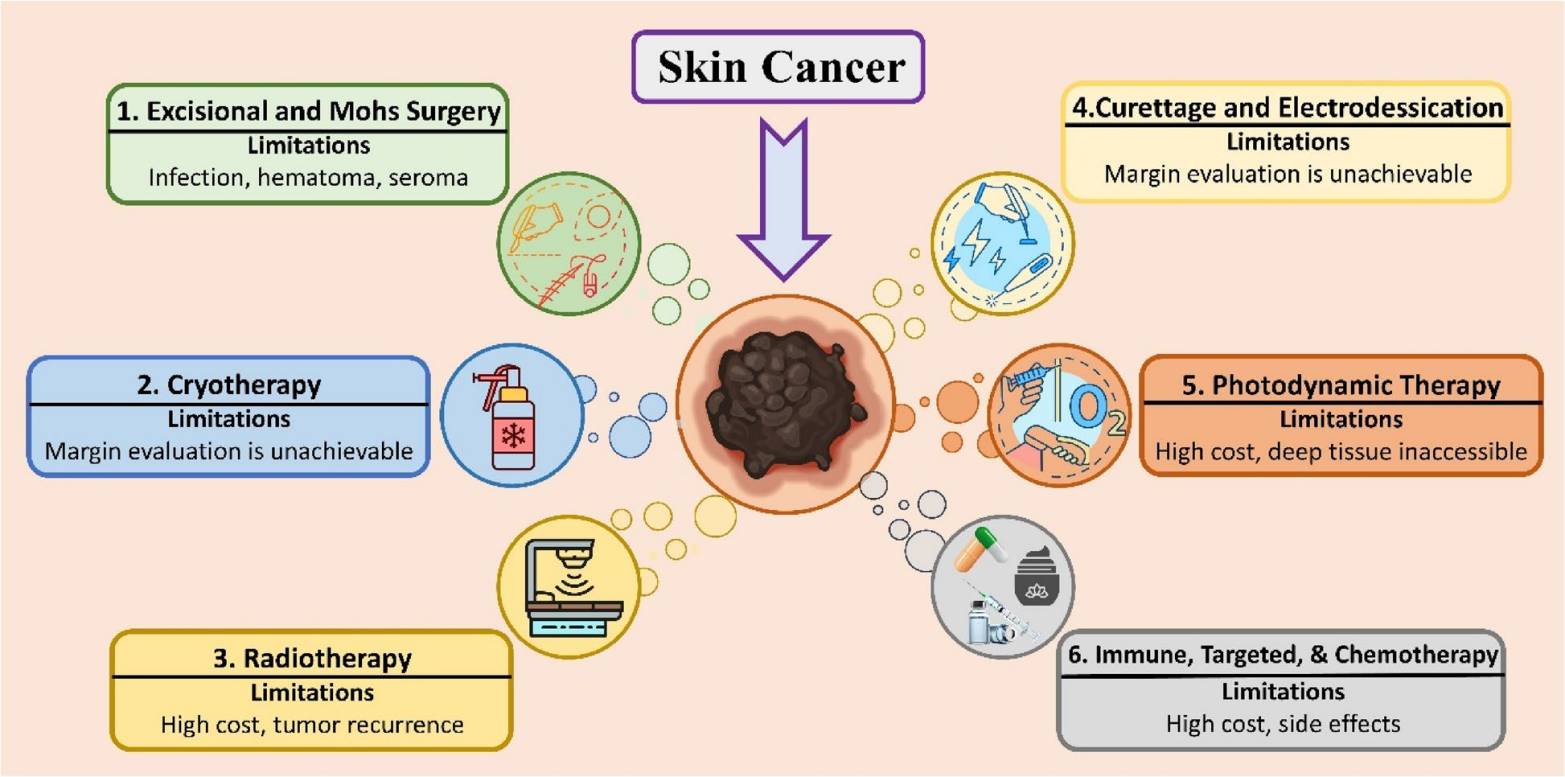
Diagrammatic representation of current treatment approaches for skin cancer and their limitations

The optimal treatment strategy for skin cancer is decided by the type, size, region, and developmental stage of the tumor [60]. Some of the regular techniques adopted to eradicate large-sized skin cancer during their initial stages are excision surgery, Mohs surgery or radiation therapy, along with immunotherapy or targeted therapy. However, small-sized skin cancer is eliminated via curettage and electrodesiccation, cryotherapy, laser therapy, or photodynamic therapy followed by immunotherapy or targeted therapy. The role of immunotherapy and targeted therapy is to make sure that the tumor doesn’t recur once they have been excised or eliminated via physical techniques. During advanced stages of skin cancer, where the tumor has metastasized into various organs like the brain, lungs, liver, or bone, chemotherapeutic agents via oral, intravenous, or topical routes are greatly recommended [61, 62]. A brief description of currently practiced treatment strategies for skin cancer therapy is narrated below (Fig. 3).

### Excisional surgery

Excisional surgery is a standard method of treating skin cancer. In this technique, the tumor is sliced every 1.5 to 2 mm in depth and processed for histopathological study. The main advantage of this technique is a negligible scar, histologic verification of tumor margin, and fast healing/recovery. However, the limitations are infection, seroma, hematoma, and the probability of significant wound formation [63, 64].

### Mohs micrographic surgery

Mohs micrographic surgery is a state-of-the-art method of excising skin tumors. In this technique, the microscope has been used to visualize and excise the maximum possible tumor under local anesthesia. It also helps in avoiding unnecessary damage to normal tissues. The horizontal sections obtained in this way furnish a complete view of the deep and peripheral margins of the specimen. Mohs surgery is more cost-effective than traditional surgical methods and impedes the recurrence of BCC or SCC [65–67].

### Curettage and electrodessication

Curettage and electrodesiccation, also called curettage and desiccation, is a specialized technique that destructs the cancer lesion and adjacent normal tissues by cauterization and also scraping with a curette. It can be implied only for small-sized skin cancers; however, it is not recommended for large and high-risk skin tumors. In addition, the margin evaluation is unachievable due to the non-availability of the specimen. Therefore, it is the least preferred technique [68–70].

### Cryotherapy

Cryotherapy is another treatment strategy that involves liquid nitrogen to freeze the small-sized BCC or SCC until they reach tumoricidal temperature. The main advantage of this technique is that there won’t be any complications of bleeding or line scar after completion of treatment, along with a high tumor clearance rate. However, due to a lack of tumor margin determination and skilled-professional dependent procedure, this technique is rarely adopted in treating skin cancer [71, 72].

### Radiation therapy/radiotherapy

Radiation therapy/radiotherapy is an ideal strategy to treat older patients with extensive and recurrent skin cancer who cannot tolerate surgery or the locations where removal of tumors is not possible surgically. This therapy is categorized into three major classes such as conventional external radiation therapy, superficial x-ray therapy, and brachytherapy. The modest technique for radiation includes volumetric arc therapy, which helps in complex dose distribution and minimizes normal tissue involvement. However, their high cost, several rounds of visits for therapy, and growth of destructive phenotypes in a few recurring tumors are some of the limitations of this therapy [73].

### Photodynamic therapy

Photodynamic therapy (PDT) is a distinctive non-invasive technique that adopts photosensitizers and lasers to kill skin cancer cells [74]. Initially, the photosensitizers are administered to make them accumulate on the tumor area, followed by irradiation of laser beam to generate singlet oxygen and other reactive oxygen species from photosensitizers, which finally kills tumor cells [75]. Some of the commonly used photosensitizers are hematoporphyrin derivative [76, 77], 5-aminolaevulinic acid [78, 79], boron-dipyrromethene [80], and so on. Studies have shown that the use of topical anticancer drugs along with PDT as a combinatorial approach is highly effective in skin tumor eradication [81]**.** The drawback associated with the technique is that high-cost and deep-rooted tumors are unable to kill effectively [82].

### Immunotherapy, targeted therapy, and chemotherapy

Immunotherapy, targeted therapy, and chemotherapy are the most promising adjuvant therapies against BCC, SCC, and melanoma [83]. Regardless of the surgery, radiation therapy, or PDT, immunotherapy, targeted therapy, or chemotherapy are highly recommended as alternative therapy for successfully curing skin cancer (advanced stage) without recurrence. Additionally, this strategy has been proven to increase the survival rate of skin cancer patients. However, the drawbacks associated with immunotherapy and targeted therapy, such as high cost and low patient compliance, are a threat [31]. Subsequently, this turns the patients’ eyes towards chemotherapy. Although chemotherapy can address the cost-related issues and makes the treatment affordable to low and middle-income families, the side-effects caused by chemotherapeutic agents and chemoresistance exhibited by the aggressive tumors are their greatest drawbacks [84, 85]. Therefore, an advanced treatment strategy that can overcome the current challenges faced by skin cancer treatment approaches is highly required to ensure patient compliance. In this quest, nanotechnology is a ray of hope for effective treatment against skin cancer.

## Nanotechnology in skin cancer therapy

**Fig. 4:**
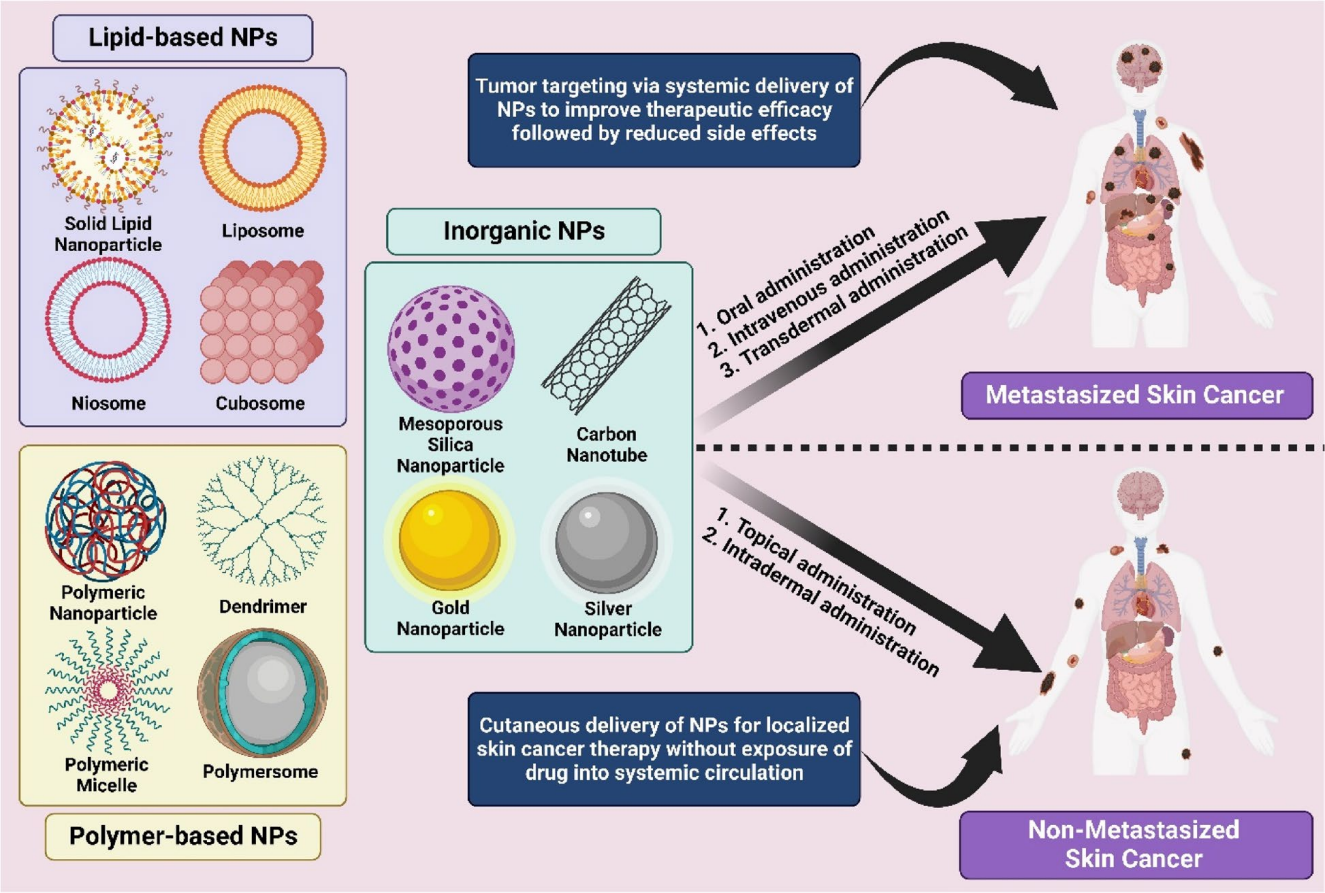
Schematic representation of utilization of nanoparticles in skin cancer therapy

Nanotechnology is an emerging area of science that involves the manipulation of various materials in the nanometre range [35, 36]. Nanomaterials have remarkable potential to improvise the performance of cancer therapeutics by acting as both drug carriers and therapeutic agents [37]. As described in section "Current treatment approaches and their limitations", the treatment for skin cancer is often chosen by the tumor type, size, region, and development stage. Regardless of surgery and radiation therapy, skin cancer is treated with immunotherapy, targeted therapy, and chemotherapy to diminish as many cancer cells as possible. However, the conventional delivery of chemotherapeutic agents lacks tumor targeting leading to inefficient tumor uptake and unnecessary distribution of drugs throughout the body, thereby causing severe side effects. In addition, the therapeutic agents that possess poor half-life, low solubility and permeability, and inadequate stability in physiological conditions fail to produce the required therapeutic efficacy [85, 86]. In most cases, where skin cancer has not been metastasized into other organs like the brain, lungs, liver, bone, etc., the direct delivery of therapeutic agents into the skin tumor site (topical) could potentially avoid the systemic toxicity along with a reduction in the overall cost of the treatment [87]. However, the sufficient permeability of the therapeutic agents into the cutaneous region of skin tumors is hindered by the skin’s outermost barrier stratum corneum. Henceforth, nanotechnology is an apt strategy to address all these issues to abolish skin cancer. There is a wide range of nanomaterials that are involved in the treatment of skin cancer conditions, among which the nanoparticles (NPs) have gained significant interest due to their unique properties, such as passive tumor targeting via enhanced permeability and retention (EPR) effect [88, 89], evading reticuloendothelial system (RES) [90], and improved skin permeability [91]. The NPs are further classified into three categories, i.e., inorganic NPs, polymer-based NPs, and lipid-based NPs (Fig. 4). The inorganic NPs are the specialized ones that perform both as drug carriers and therapeutic molecules [92], whereas polymer and lipid-based NPs are well suited for delivering therapeutic molecules of various kinds in a controlled manner with enhanced permeability (through the skin and other tissues including tumors) [93, 94].

## Inorganic nanoparticles for skin cancer therapy

Inorganic NPs have grasped significant attention in oncology for their diverse applications like tumor therapy, tumor drug delivery, tumor imaging, and enhancement of radiotherapy. These NPs are derived from metals, metal oxides, carbon, ceramics, silica, etc. The unique physicochemical properties of inorganic NPs, including small size, large surface area, bioactivity, biocompatibility, and functionalizing ability, have made them the most appropriate candidates for skin cancer therapy. Scientists have explored that the inorganic NPs possess the intrinsic therapeutic property, due to which they can execute the cancer cells by themselves [95, 96]. In addition, they can also deliver a wide range of therapeutic agents to tumor sites via active or passive targeting. Nevertheless, they can play the role of photothermal or photosensitizing agent, which is further employed in photothermal or photodynamic therapy (PTT/PDT), respectively [95]. Together with intrinsic therapeutic property, drug delivery ability, and photothermal or photosensitizing trait, the inorganic NPs can endow exceptional synergistic treatment for skin cancer. A few regular inorganic NPs involved in skin cancer therapy are mesoporous silica NPs, gold NPs, carbon nanotubes, silver NPs, platinum NPs, zinc oxide NPs, copper oxide NPs, titanium dioxide NPs, cerium oxide NPs, and so on. Further, the most recent studies of these NPs against skin cancer have been thoroughly described in the coming sections.

### Mesoporous silica nanoparticles

Mesoporous silica nanoparticles (MSNs) are a unique type of NPs distinguished by repeated positioning of uniform-sized mesopores whose pore diameters range from 2 to 7 nm placed in an organized order of silica with an average diameter ranging from 50 to 300 nm as per the International Union of Pure and Applied Chemistry (IUPAC) [97, 98]. MSNs were first developed by the scientists of Mobil corporation in the year 1992 via a liquid crystal template mechanism using aluminosilicate gels as a precursor [99]**.** The general mechanism behind the formation of MSNs involves supramolecular assemblies of surfactants to form micelles at a concentration higher than the critical micelle concentration (CMC), followed by condensation of silica precursors on the surface of micelles, which leads to the formation of inorganic–organic hybrid system. Thereafter, the template surfactant can be eliminated by calcination or solvent extraction to form mesopores [100]. The obtained MSNs can offer a wide range of biomedical applications due to their unique properties such as uniform porous structure, large specific surface area, pore volume, tuneable particle size, dual functional surfaces (inner porous surface and outer matrix surface), and good biocompatibility and biodegradability. Some of the most significant advantages of MSNs in cancer therapy are their high drug loading capacity, enhanced skin permeability (by functionalizing with polymers and peptides), non-premature release and safeguarding of therapeutics from degradation in unfavorable physiological conditions, controlled release of therapeutic agents through modification with stimuli-responsive materials, passive targeting of tumors via EPR effect, and active targeting of tumors via ligand-functionalization [101, 102]. Owing to this supremacy, the MSNs can be considered exemplary nanosystems that could actively participate in skin tumoral therapy.

Cisplatin (CP) is a potent chemotherapeutic agent with several drawbacks such as nephrotoxicity, ototoxicity, hepatotoxicity, acquired tumor resistance, etc. [103]. In order to diminish its toxicity toward normal cells and increase its anticancer effectiveness, SBA-15 (Santa Barbara Amorphous 15) based MSNs impregnated with CP were developed by Draca and colleagues [104]. The results from MTT assay revealed that the CP@MSNs possess an IC_50_ value of 0.58 ± 0.11 µM, which was lesser than the IC_50_ value of free CP (0.72 ± 0.17 µM). In in vivo study, the free CP did not inhibit even 5% of tumor growth, whereas CP@MSNs substantially declined the tumor size. The authors also confirmed that the increased antitumoral effect of CP@MSNs is purely because of the encapsulated CP and not due to the MSNs, thereby proving MSNs are inactive drug carriers. In addition, the mice group treated with free CP lost their body weight significantly (10–15%) and indicated several side effects such as heavy breathing, aggravated moving, vocalizations, etc. However, no side effects were observed in the mice group treated with CP@MSNs apart from mild to negligible nephro- and hepatotoxicity, which did not affect the mice to a greater extent, ensuring MSNs are the prominent candidates in effective melanoma treatment without involving severe side effects.

**Fig. 5:**
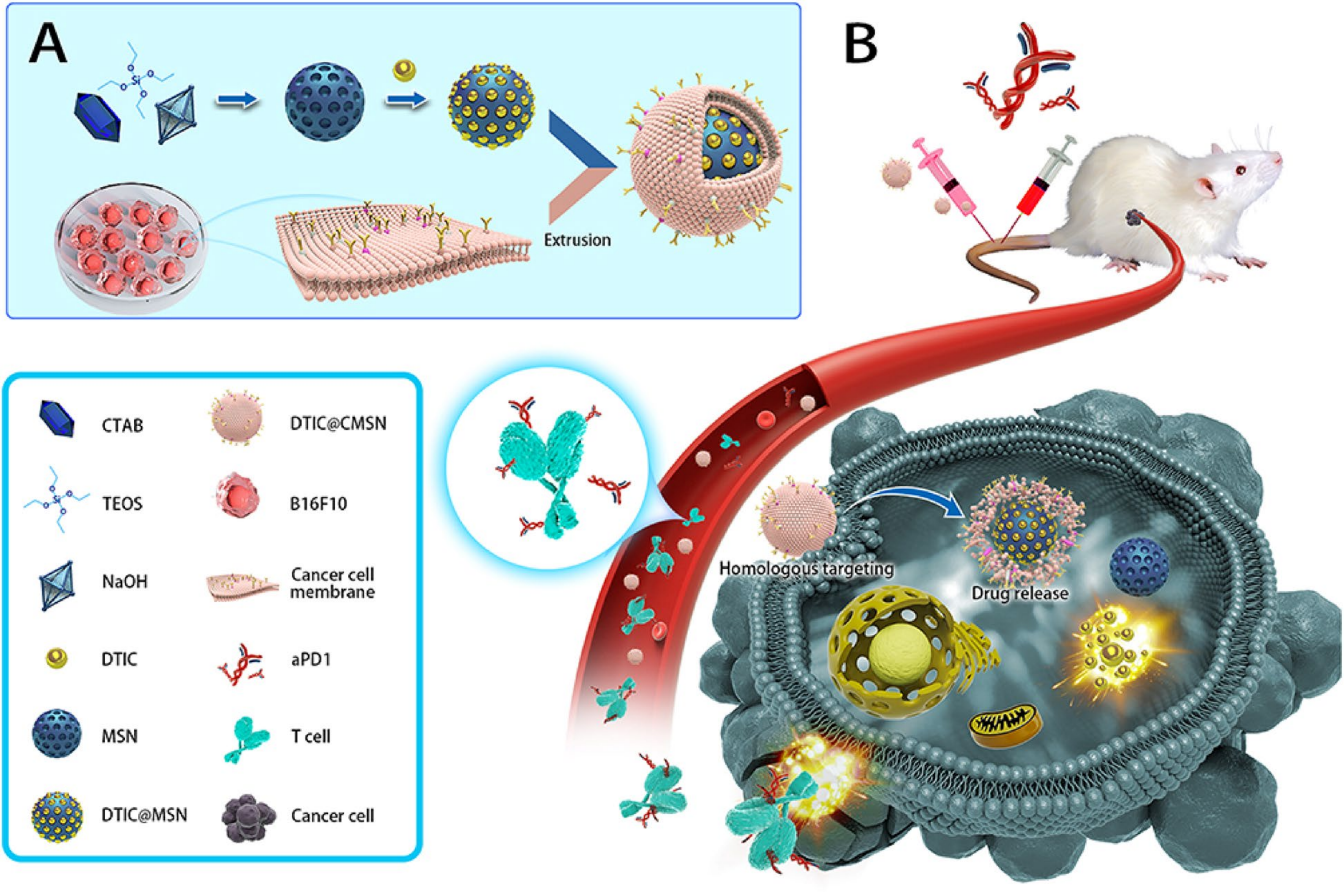
**A** Diagrammatic representation of dacarbazine (DTIC) imbibed cancer cell membrane camouflaged mesoporous silica nanoparticle synthesis process (DTIC@CMSN). **B** Schematic illustration of antitumor immune response induced by DTIC@CMSN merged with anti-programmed cell death protein 1 antibody (aPD1), reproduced with permission from [107], licensed under CC BY 4.0

Dacarbazine (DTIC) is the only drug approved by the USFDA since 1975 as a first-line chemotherapeutic agent for the treatment of melanoma [105]. However, it bears certain drawbacks such as extreme sensitivity to light and temperature, highly cytotoxic in normal cells, unstable in solution form (used as drug powder injection), and poor half-life; due to which, the overall response rate of DTIC in patients with advanced stage of melanoma was found to be only 5–20% [106]. Therefore, a recent study by Zhao and colleagues developed DTIC@MSNs with a particle size of 142 nm in the quest to overcome the drawbacks associated with free DTIC [107]. Although the DTIC@MSNs possess an advantage over free DTIC, such as enhanced tumor uptake via the EPR effect, less than 1% of DTIC@MSNs reach the tumor site via a passive targeting strategy. This opens the door for active targeting of NPs using various targeting moieties such as aptamers, peptides, and antibodies. However, it is a tedious process due to the involvement of multiple chemical reactions. Thus, the authors came up with the idea of coating cancer cell membrane (CCM) on DTIC@MSNs via extrusion method that resulted in a particle size of 151 nm (Fig. 5). This benefited the nanosystem by lowering the systemic clearance (RES uptake) and increasing the targeting ability, thereby resulting in accumulation of most DTIC@CMSNs inside the tumor. Further, the coated CCM also furnished extra protection to DTIC from leakage before entering inside the tumor. The melanoma cancer cell lines (B16F10) treated with DTIC@CMSNs induced 40% of cell death, twice as compared to free DTIC, which caused only 20% of cell death. Finally, the authors used anti-programmed cell death protein 1 antibody (aPD1) along with DTIC@CMSNs to mitigate the immune’s negative feedback pathway throughout the action of chemotherapeutics (Fig. 5). Overall, DTIC@CMSNs combined with aPD1 exhibited both improved tumor inhibition and declined systemic adverse reactions, making them interesting candidates in melanoma therapy.

Nowadays, herbal constituents are gaining significant attention in cancer therapy due to their ability to not cause any potential side effects. One such phytoconstituent is resveratrol (RVT), which has shown promising results in cancer therapy [108]. However, its efficacy is hindered by poor solubility in aqueous medium. To overcome this issue, the RVT was loaded into MSNs by Marinheiro and team to treat the melanoma condition [109]. The particle size and drug entrapment efficiency of developed RVT@MSNs were found to be 60 nm and > 93%, respectively. The loading of RVT into MSNs enabled amorphization, due to which the solubility of RVT@MSNs is substantially improved than free RVT. Exhibiting the pH-dependent drug release (pH 5.2), the RVT@MSNs were found to be a suitable delivery system in the tumor microenvironment. Further, the RVT@MSNs exhibited improved cytotoxicity in two different melanoma cell lines (A375 and MNT-1) compared to free RVT. However, preclinical studies need to confirm these results further to accept RVT@MSNs as a suitable system for melanoma treatment.

Most of the therapeutic agents that are used in the treatment of skin cancer are administered through an intravenous route. However, this could lead to unnecessary distribution of drug throughout the body, increasing the dose required to exhibit minimum therapeutic efficacy. As a solution to this issue, researchers came up with dermal/transdermal drug delivery systems for the localized and site-specific delivery of therapeutics into the skin tumors. However, the permeability of therapeutic agents (molecular weight more than 500 Da, highly lipophilic and hydrophilic) through the skin has remained the biggest challenge (due to stratum corneum) [87]. Thus, a study by Lio and colleagues developed small interfering RNA (siRNA) (10–20 kDa) loaded MSNs for treating squamous cell carcinoma (SCC) via transdermal route [110]. Initially, the authors loaded molecular beacon (MB) into MSNs as a model drug instead of siRNA to optimize the formulation. The MB-loaded MSNs had an average particle size of 200 nm and 4 nm mesopore size. The developed NPs were negatively charged due to the inherited negative charge of MB. However, studies have depicted that positively charged NPs possess greater affinity towards negatively charged skin pores, when applied on untreated skin. Therefore, the authors coated MB@MSNs with positively charged poly-L-lysine (PLL) and further confirmed the charge with zeta potential study that exhibited + 30 mV. Due to the coating of PLL, the size of MB@MSNs-PLL was increased from 200 to 250 nm. The biodistribution study using a model drug (Cy5) indicated that maximum concentration of drug accumulated on tumor site after administering via intratumor injection followed by topical application (Aquaphor® as a vehicle), lastly intravenous injection. It was also found that the NPs administered topically yielded less distribution of Cy5 in all chief organs (liver, heart, kidney, lung, and spleen) compared to intratumor and intravenous injection. Finally, the topically delivered siRNA@MSNs-PLL exhibited the highest rate of tumor inhibition in the mouse xenograft model (SCC) compared to intratumor and intravenous injection proving that MSNs in combination with topical delivery is a promising approach for the efficient treatment of SCC.

PTT sought to serve as an essential modality in cancer therapy due to its fantastic feature of transforming the energy of near-infrared light (NIR) into thermal energy with the help of distinctive photothermal agents [111]. However, the anticancer efficacy can still be improved if PTT is combined with chemotherapy. In this view, Zhang and co-workers developed manganese-doped MSNs loaded with indocyanine green (ICG) (NIR dye) and DTIC (chemotherapeutic agent) to treat malignant melanoma [112]. The particle size of MSNs was found to be 154 nm with a 3.3 nm pore size. Further, the results from in vivo study exhibited maximum tumor reduction in the nude mice group treated with ICG/DTIC@MSNs + NIR irradiation (808 nm, 10 min) compared to free DTIC, ICG@MSNs + NIR irradiation, ICG/DTIC@MSNs. These results show hope that chemo-photothermal therapy is a promising treatment modality in melanoma therapy without significant side effects.

Some of the studies that demonstrated promising results in skin cancer therapy include Verteporfin/ MSNs/ melanoma [113], indomethacin/ MSNs, 3-aminopropyltriethoxysilane alkoxide/ melanoma [114]**,** curcumin/ MSNs, PEG-400/ melanoma [115]**,** ruthenium (II)/ MSNs, (2-thienylmethyl) hydrazine hydrochloride (H1), (5,6-dimethylthieno[2,3-d] pyrimidin-4-yl) hydrazine/ melanoma [116]**,** Verteporfin/ MSNs, aminopropyltriethoxysilane/ melanoma [117]**,** 5-fluorouracil, dexamethasone/ MSNs, 3-aminopropyltriethoxysilane/ melanoma [118]**,** siRNA/ nucleic acid NPs, MSNs/ melanoma [119]**,** HGP10025–33, TRP2180–188/ MSNs/ melanoma [120]**,** ovalbumin/ MSRs, MSNs/ melanoma [121]**,** polydopamine, ovalbumin/ MSNs, ammonium bicarbonate/ melanoma [122]**.**

### Carbon nanotubes

Carbon nanotubes (CNTs) are cylindrical nanostructured carriers constructed by rolling of graphene sheets [123]. They were first reported by a Japanese physicist named Sumio Iijima in the year 1991 [124]. The CNTs formed by a single sheet of graphene are termed single-walled CNTs (SWCNTs), whereas several graphene sheets roll up to yield multi-walled CNTs (MWCNTs). Although the diameter of both SWCNTs and MWCNTs lies in the nm range, their length can extend up to several mm. The CNTs are estimated to be apt candidates for cancer therapy due to their distinct structural, mechanical, electrical, and thermal properties (PTT). The large surface area of CNTs allows them to load high concentration of anticancer therapeutics either by using disulfides as linkers or via adsorption, and further the controlled drug delivery can be achieved through modification of CNTs with stimuli-responsive materials [125, 126]. Studies have also explored the skin permeability potential of CNTs to deliver therapeutic agents via the transdermal route. But it has been found that the CNTs alone cannot permeate through the skin. However, few studies have reported the improved skin permeability of CNTs under lipid/polymer functionalization and iontophoresis [127]. All these evidences motivate biomedical researchers to explore their potential in skin cancer therapy.

Besides their therapeutics delivery ability and photothermal property, the CNTs also possess intrinsic anticancer properties. In a study, Naserzadeh and team compared the antimelanoma efficacy of SWCNTs and MWCNTs, followed by exploring the mechanism by which they kill melanoma cells [128]. From the in vitro results, it has been found that SWCNTs are more cytotoxic than MWCNTs in melanoma cell lines. This may be due to the smaller size of the SWCNTs. Interestingly, the antimelanoma activity CNTs was due to the activation of caspase 3 through mitochondria pathway followed by ROS generation, which finally leads to mitochondrial membrane potential decline and cytochrome c release leading to melanoma cell death.

Another study by a Spain-based research group led by Fanarraga demonstrated the mechanism of antimelanoma activity of MWCNTs [129]. Astonishingly, it has been found that the MWCNT filaments translocate inside the melanoma cells and intermingle with the protein nanofilaments of the cytoskeleton, obstructing with the biomechanics of melanoma cell division, leading to its death. The exact mechanism is being followed by the traditional microtubule-binding anticancer agents such as paclitaxel (PTX). What is more interesting is that these MWCNTs can induce antitumoral activity even in PTX-resistant melanoma cells, making them one of the groundbreaking therapeutics carriers cum antimelanoma agents exhibiting potential synergistic activity.

Myeloid-derived suppressor cells (MDSC) are a heterogeneous group of immature myeloid cells that possess potent immune suppressive abilities leading to tumor progression. However, depletion of MDSC was found to have a direct relationship with the potential inhibition of tumor growth [130]. Thus, targeting MDSC with specific chemotherapeutic agents to promote apoptotic cell death is a forefront strategy. Nevertheless, conventional delivery of chemotherapeutic agents is associated with severe toxicity and hypersensitivity reactions. In this situation, Burkert and co-workers developed PTX-loaded cup-shaped carbon nanotubes doped with nitrogen (NCNC) and stoppered with gold NPs for passive tumor-targeted delivery to deplete the active MDSC [131]. The developed carbon nanotube cups enzymatically open via degradation of carbon-based material to deliver the loaded PTX at the tumor site with the help of nitrogen and reactive oxygen species produced by MDSC. The TEM results indicated that PTX@Au-NCNC possess a length of 550 ± 260 nm along with a width of 55 ± 17 nm. Since the MDSC predominantly expresses the oxidative biodegradation reagents, the authors expect these nanosystems to disintegrate in MDSC that are circulating and located in lymphoid tissue instead taken up by the tumor microenvironment via EPR effect. Finally, the results from in vivo study indicated maximum tumor growth inhibition in the mice group (melanoma bearing C57BL/6 mice) treated with PTX@Au-NCNC compared to empty Au-NCNC and free PTX. Furthermore, it is interesting to observe that the empty Au-NCNC has suppressed tumor growth better than free PTX. This could be due to the inherent antitumor properties of gold and CNTs.

To overcome the drawbacks of passive targeted drug delivery, a study by Das and colleagues reported the fabrication of curcumin (CUR) loaded SWCNTs attached with α5β1 integrin receptor targeting RGDK (Arg-Gly-Asp-Lys) tagged lipopeptide for targeted delivery of CUR to melanoma [132]. The TEM images of aqueous SWCNTs dispersion revealed that the diameter and length of CNT are around 3–5 nm and 300–500 nm, respectively. In an in vitro cell line study, it has been found that the CUR@RGDK-SWCNTs exhibited declined B16F10 cell viability compared to free CUR. After 24 h of IV injection, the maximum accumulation of CUR@RGDK-SWCNTs was found at the tumor site than in other major organs such as the spleen, heart, lung, kidney, and liver supporting the tumor-targeting ability of CUR@RGDK-SWCNTs. Thus, this nanosystem could find promising applications in melanoma therapy, specifically in delivering potent hydrophobic anticancer drugs selectively to the tumor tissues.

The CNTs are promising candidates in PTT due to their ability to absorb NIR as well as their strong photothermal conversion efficiency. However, the intravenously administered free CNTs lack tumor targeting ability. Thus, in an exciting study, Nagai and teammates reported the fabrication of SWCNTs conjugated with anti-TRP-1 (melanoma targeting moiety) using maleimide chemistry for targeted PTT without impeding the NIR absorption characteristics of SWCNTs [133]. Interestingly, in another study, Wang and colleagues developed the MWCNTs individually loaded with both chemotherapy (doxorubicin; DOX) and immunotherapy (oligodeoxynucleotides containing CpG motifs; CpG ODN) agents for combinatorial photothermal and chemo-immunotherapy of melanoma [134]. The diameter CpG@MWCNTs and DOX@MWCNTs were found to be 197.3 ± 5.45 nm and 263.8 ± 7.36 nm, respectively. Together with the intratumor injection of both CpG@MWCNTs and DOX@MWCNTs followed by NIR irradiation, the maximum antitumor activity in C57BL/6 mice bearing melanoma was witnessed compared to individual treatment approaches. All these studies suggest that CNTs are noteworthy candidates to take part in skin cancer treatment.

AgNPs, MWCNTs, PEG1000/ melanoma [135]**,** AgNPs, MWCNTs/ melanoma [136]**,** MWCNTs/ melanoma [137]**,** MWCNTs/ melanoma [138]**,** phenylboronicacid, trimesic acid, SWCNTs/ melanoma [139] are few of the recent investigations for the treatment of skin cancer.

### Zinc oxide nanoparticles

Zinc is a transition metal that is a key and profuse trace component in the body following iron. It is a pivotal component in diverse cell functions and displays its significant part in supporting cellular homeostasis [140]. Zinc oxide NPs (ZnO NPs) have taken part in many biomedical applications due to their inherent nutritional benefits and relatively low toxicity compared to other metallic NPs. Owing to their large surface area to volume ratio and small particle size (less than 100 nm), the ZnO NPs possess inherent cytotoxicity behavior against cancer cells. So far, the most widely reported mechanism behind the anticancer activity of ZnO NPs is their ability to produce a large number of reactive oxygen species after entering the tumor cells. Thanks to the semiconductor property of ZnO NPs, which is a crucial factor behind the production of ROS, resulting in cancer cell death via apoptosis. ZnO NPs were also found to take part in both PTT/PDT [141–143]. Further, they can be functionalized with various polymers and peptides to achieve active tumor targeting, enhanced skin permeability (cutaneous skin tumor targeting), and also can be conjugated with numerous therapeutic agents to acquire synergetic anticancer activity. Additionally, the larger ZnO is being considered as Generally Recognized as a Safe component by FDA, making them the safe and appropriate choice for skin cancer therapy.

Recently, a study reported by Khan and co-workers involved the development of ZnO NPs using cetyltrimethylammonium bromide (CTAB) (capping agent) and varying concentrations of ion-carriers (NaOH) to study their physicochemical and biological properties [144]. The SEM images displayed that both the NPs were in spider chrysanthemum-like shape. The particle size by TEM images revealed 40 nm for ZnO NPs-1 (0.01 M NaOH) and less than 20 nm for ZnO NPs-2 (0.005 M NaOH). The in vitro cytotoxicity study using human epidermoid carcinoma A431 cells (non-melanoma) showed increased cell viability in ZnO NPs-1 treated group, concluding that ZnO NPs-2 are more cytotoxic. Furthermore, the ROS generation and caspase-3 activity was found to be higher in ZnO NPs-2 treated group as compared to ZnO NPs-1, concluding that smaller-sized ZnO NPs exhibit enhanced cytotoxicity against non-melanoma human cell line (A431). These NPs need to be further studied in preclinical settings to clarify their antimelanoma properties.

Ras proteins mutations are usual in almost all types of cancers, including skin cancer [145]. Ras proteins have a principal role in regulating different cellular signaling pathways, due to which they are the targets for intracellular delivery of the Ras binding domain (RBD) [146]. However, due to the lack of penetrating ability of free RBD into tumor cells, there is a need for a delivery system that can enhance the anticancer activity of RBD. Therefore, Mathew and team devised a strategy to improve the antimelanoma efficacy of RBD by conjugating it with ZnO NPs [147]. The particle size of plain ZnO NPs was found to be 14 nm; however, after attaching it with RBD, the size increased to 100 nm. The in vitro cytotoxicity study on mouse melanoma cell lines displayed increased cell death for RBD@ZnO NPs (100 nm) than free RBD and ZnO NPs (14 nm). The promising in vitro results further demand investigation in preclinical settings.

Oxidative stress in any cells, including cancer, is avowed to cause malfunction of cell organelle via membrane disruption, mitochondrial dysfunction, or Golgi and deoxyribonucleic acid fragmentation [148]. In this quest, Ghaemi and co-workers developed the Ag@ZnO NPs to use as a photosensitizer that can generate increased ROS inside the melanoma cells leading to its death upon UV irradiation (PDT) [149]. In this study, the authors intended to foster the damage of organelle followed by the arrest of melanoma cell cycle via boosting the ROS level intracellularly, resulting in apoptosis and autophagy. The in vitro cell line studies revealed that the Ag@ZnO NPs + UV (290–320 nm, 450 W lamp, 40 cm field-focus distance, 180 s exposure time) were highly cytotoxic in A375 human melanoma cell lines. In contrast, they remained unaffected in normal dermal fibroblast cell lines. All these evidences encourage Ag@ZnO NPs to be a promising PDT agent to eradicate cutaneous melanoma.

The siRNA and microRNA (miRNA) are widely reported in cancer therapy for targeted hindrance of cancer protein translation [150]. Unfortunately, they are meant to suppress the function of one gene at once. However, a polyinosinic-polycytidilic acid (pIC) (RNA with double strand) possess both immunogenic and anticancer property [151]. Further, the surface functionalized NPs were widely used to deliver this RNA molecule to the tumor site. But, for the first time, a study by Ramani and team directly attached the pIC on top of ZnO NPs to form RNA corona around the surface of NPs without involving any surface modifying agents to treat melanoma [152]. The pIC RNA-bound naked ZnO NPs possess synergistic antimelanoma activity due to the dual inherent anticancer property of both pIC RNA and ZnO NPs. The particle size of plain ZnO NPs and pIC@ZnO NPs were found to be 60–70 nm and 200–240 nm, respectively. The developed nanosystem exhibited efficient antimelanoma activity both in in vitro (B16F10 and A375 cell lines) and in vivo (melanoma bearing BALB/c mice) conditions. This makes them the most unambiguous agents for melanoma therapy.

So far, we have come across various studies involving ZnO NPs for different purposes in skin cancer therapy, such as chemotherapeutic agents’ delivery, photothermal agent for PTT, photosensitizer for PDT, inherent anticancer agent, biomolecules delivery, stimuli-responsive therapeutics delivery, and so on. In an exciting study, Zhang and colleagues developed a chemotherapeutic agent (DOX) loaded on mesoporous silica-coated gold NPs that is finally capped with ZnO quantum dots (QDs) [153]. It is a 4-in-1 nanosystem that performs as a (i) photothermal agent due to the presence of gold NPs, (ii) loads DOX due to the suffice pores on coated mesoporous silica, (iii) delivers DOX in a pH-responsive manner due to the gatekeeping characteristics of ZnO QDs, (iv) further possess the inherent anticancer property of ZnO QDs. The particle size of initial gold NPs was found to be 18 nm, that further increased to 72 nm after forming AuNP@mSiO2 with a pore size of 2.8 nm. On the other arrow, the ZnO QDs exhibited a particle size of 5 nm. However, the authors do not disclose the overall size of AuNP@mSiO2@DOX-ZnO nanosystem. The developed AuNP@mSiO2@DOX-ZnO nanosystem exhibited 60% DOX release in pH 5.0 buffer system (acetate), whereas only 8% DOX release was observed in pH 7.4 buffer system (phosphate), indicating the tumor pH-responsive drug delivery. Further, the melanoma-bearing C57BL/6 mice treated with AuNP@mSiO2@DOX-ZnO + laser irradiation (L) displayed the highest tumor growth inhibition and lung metastasis suppression with no significant side effects such as tissue damage and loss of body weight. The findings suggest that AuNP@mSiO2@DOX-ZnO would be a favorable nanosystem for the combined treatment of melanoma.

Few more studies that exhibited favourable results against skin cancer include ZnO NPs/ *Musa sapientum*/ squamous cell carcinoma [154]**,** ZnO-CuO NPs/ *Sambucus nigra L*/ melanoma [155]**,** ZnO NPs/ *Alpinia calcarata*/ squamous cell carcinoma [156]**,** ZnO NPs/ *Bacillus cereus* PMSS-1/ melanoma [157]**.**

### Gold nanoparticles

Gold, in its colloidal form, has taken part in numerous medicinal applications for centuries. The first scientific piece of work on gold NPs (AuNPs) was presented in 1857 by Faraday. Since then, several studies have been conducted to explore their biomedical applications. Among many, cancer therapy is one of the appealing areas where efficient and cost-effective treatment is in urgent need [158, 159]. AuNPs have gained much attention on the other arrow due to their easy, inexpensive, and reliable synthesis methods. Studies have shown that the nano-sized gold particles (less than 100 nm) are highly efficient in selectively targeting and uptake into the tumors [160]. The AuNPs were also reported to inhibit angiogenesis, which is a critical factor in tumor development. So far, the most widely accepted mechanism for inhibition of angiogenesis is the interaction of AuNPs with the heparin-binding growth factors such as vascular permeability factor/vascular endothelial growth factor (VPF/VEGF)-165 and basic fibroblast growth factor (bFGF) thereby inhibiting their activity. This hampers endothelial/fibroblast cell proliferation via depleting the phosphorylation rate of angiogenesis accountable proteins [161, 162]. Additionally, the AuNPs can be effectively used in PTT through their surface plasmon resonance (SPR) effect. Their strong optical absorbance permits constructive laser therapy against tumors with negligible collateral damage to the neighboring healthy tissues [163]. Nevertheless, the AuNPs can be functionalized with various polymers, peptides, and therapeutic agents to achieve active targeting of tumors, enhanced skin permeability (cutaneous skin tumor targeting), controlled delivery of therapeutics, and synergistic activity against cancer cells. With respect to all these merits, AuNPs could be considered a decorous aspirant in treating skin cancer.

Generally, CTAB, a positively charged surfactant, is used as a stabilizer in the synthesis of AuNPs, which is deemed cytotoxic. Therefore, a recent study by Goncalves and teammates synthesized the gum-arabic coated gold nanorods (GA-AuNRs) for treating aggressive melanoma conditions without severe toxicity to normal cells [164]. The GA is a negatively charged polysaccharide that selectively binds and encapsulates the CTAB electrostatically. The TEM micrographs displayed that the resulting GA-AuNRs were in the transversal size of 24.5 ± 6.1 nm and longitudinal size of 48.3 ± 6.6 nm. In normal fibroblast cell lines, the GA-AuNRs exhibited 30% less cytotoxicity than CTAB-AuNRs. However, slightly increased toxicity in melanoma cell lines was witnessed for GA-AuNRs than CTAB-AuNRs. Further, the in vivo study on melanoma-bearing mice model depicted significant tumor growth inhibition in a concentration-dependent fashion. All the findings conclude that the intrinsic property of AuNRs coated with negatively charged GA is a noteworthy candidate to participate in combinatorial antimelanoma therapies to explore their synergistic potential.

Angiogenesis enacts a primary part in tumor development and its metastasis. VEGF-A and VEGF receptor-2 (VEGFR-2) are two chief factors in the progression of angiogenesis. Sorafenib (Sor) is a multi-kinase inhibitor that has a demonstrated history of targeting VEGFR, platelet derived growth factor receptor (PDGF), and Raf to inhibit tumor progression [165]. However, the drawbacks of Sor, such as poor solubility, rapid metabolism, and low bioavailability, hinder them from exhibiting complete action. Therefore, Huang and team investigated the effect of Sor derivatives capped AuNPs on melanoma inhibition [166]. The synthesized AuNPs and Sor-AuNPs revealed a particle size of 58.2 ± 7.1 nm and 337.9 ± 13.0 nm, respectively, as confirmed by both DLS and TEM. Further, in the melanoma-bearing mice model, the orally administered Sor-AuNPs exhibited maximum antitumoral activity than free Sor displaying the AuNPs could be potential carriers of Sor in antimelanoma therapy.

**Fig. 6:**
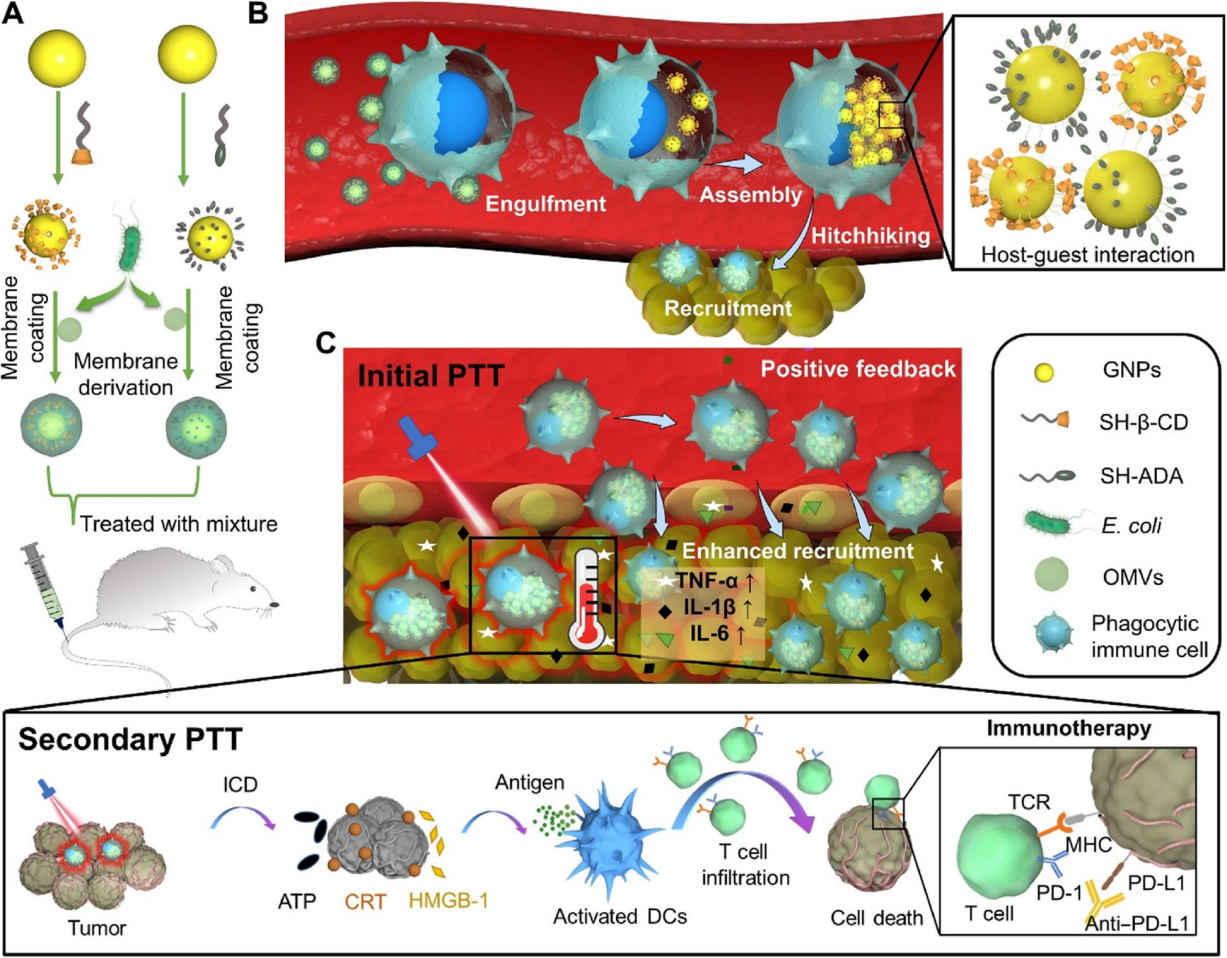
In vivo construction of immune cell-based nanomedicine carriers and initial PTT treatment enhance hitchhiking delivery into the tumor and improve antitumor immunotherapy. **A** E. coli OMVs are coated on both CD-GNPs and ADA-GNPs to prepare bacteria-mimetic nanoparticles. **B** Selective phagocytosis of bacteria-mimetic nanoparticles by phagocytic immune cells induces OMV degradation and subsequent intracellular aggregation of GNPs mediated by CD-ADA host–guest interactions, leading to photothermal property due to the plasmonic effects of GNP aggregates. The large size of intracellular GNP aggregates also inhibits the leakage during in vivo cell-hitchhiking delivery. Because of the inflammatory tropism to melanoma, immune cells achieve the targeted delivery of intracellular GNP aggregates to the tumor tissues. **C** Initial PTT treatment of GNP aggregates induces tumor damage that subsequently enhances inflammatory signals and provides positive feedback to recruit more immune cells (including the carriers) for enhanced antitumor therapy. Secondary photothermal treatment (PTT) of Mixture induces tumor cell immunogenic cell death (ICD) and activates antitumor immune response, further strengthened by immune checkpoint blockage (aPD-L1), reproduced with permission from [168], licensed under CC BY 4.0

Recently, cell-based drug carriers have emerged due to their ability to selectively target the tumor and deliver anticancer therapeutics without any adverse effects [167]. However, the immunosuppressive behavior of the tumor microenvironment indeed results in inefficient uptake of immune cell-based systems into the tumor. In order to find a solution to the above problem, Gao and co-workers reported a unique technique to stably hitchhike phagocytic immune cells through specific phagocytosis of bacteria-imitating AuNPs followed by concurrent self-assembly via a supramolecular mechanism inside the cancer cell (Fig. 6) [168]. In this study, the authors have developed β-cyclodextrin (β-CD) attached AuNPs and adamantane (ADA), followed by coating with vesicles formed by the outer membrane of *E. coli* bacteria (OMVs). The coated OMVs induced phagocytosis of AuNPs via intracellular degradation and supramolecular self-assembly of AuNPs accelerated by β-CD@ADA interactions. Once the AuNPs were accumulated inside the tumor by phagocytic immune cells, the PTT treatment induced enhanced tumor damage and also accelerated the accumulation of AuNPs aggregates inside the tumor. This strategy evidenced the effective antimelanoma PTT/immunotherapy via a unique bacteria-imitating nanosystem, making them a promising candidate for further clinical studies.

Anti-programmed cell death protein-1 (anti-PD-1) immunotherapy is considered to be an efficient treatment strategy against melanoma [169]. However, tumor resistance to such immunotherapy hinders their therapeutic efficacy. Conversely, miRNAs have gained significant interest in tumor growth suppression via ferroptosis. Altogether, to enhance the effectiveness of anti-PD-1 and to improve antimelanoma activity, Guo and team developed the miR-21-3p-loaded AuNPs and further closely studied its effect on anti-PD-1 immunotherapy in melanoma mice models [170]. The results from DLS revealed that the miR-21-3p@AuNPs were in the size range of 70–100 nm with a zeta potential of 0 mV (indicates highly unstable in solution form). Further, it has been found that the miR-21-3p upregulation significantly enhanced the efficacy of anti-PD-1 via inducing lipid peroxidation and suppressing TXNRD1 gene that ultimately leads to melanoma cell ferroptosis. Witnessing this, the AuNPs conjugated miR-21-3p could be a promising system to increase the efficacy of immunotherapy in the treatment of melanoma conditions.

Similarly, other investigations that have endowed a ray of hope for efficient skin cancer treatment include AuNPs, AD-Acp-FFRKSIINFEKL/ β-cyclodextrin/ melanoma [171]**,** AuNPs/ *Cassia fistula*, human serum albumin/ melanoma [172]**,** AuNPs/ L-ascorbic acid, hyaluronic acid, oleic acid/ melanoma [173]**,** Au-silica core shell, glucosamine/ mercaptoecanoic acid, N-hydoxysulfosuccin imide/ melanoma [174]**,** AuNPs/ *Tasmannia lanceolata, Backhousia citriodora*/ melanoma [175]**,** curcumin, AuNPs/ red blood cell membrane, platelet membrane/ melanoma [176]**,** AgNPs/ oligonucleotides, PEG(polethylene glycol)800-SH/ melanoma [177]**,** AuNPs/ melanoma [178]**,** AuNPs/ cysteamine, folic Acid/ melanoma [179]**,** AuNPs/ sodium citrate/ melanoma [180]**,** AuNPs/ SH- PEG-COOH, cetyl trimethylammonium bromide/ melanoma [181]**.**

### Silver nanoparticles

Silver NPs (AgNPs) have attracted increasing interest as potential anticancer agent due to their physicochemical properties, such as small particle size, high conductivity, chemical stability, and surface plasmon resonance (used in PTT) [182]. The biological activity of AgNPs has been attributed to the presence of the silver ion. The small-sized AgNPs (less than 100 nm) tend to utilize the leaky vasculature of tumors to enter inside at maximum concentration. AgNPs have demonstrated exceptional antitumoral activity by inducing oxidative stress inside the cancer cells and also by using the energy provided by glucose in the media. Studies have reported that the most common mechanism by which the AgNPs exhibit anticancer activity were apoptosis, autophagy, and anti-angiogenesis (VEGF-induced angiogenesis only) [183, 184]. Further, the skin penetration ability of AgNPs is lower than other metallic NPs, such as gold, since a large percentage of free ions are precipitated as silver-sulfide in the skin’s outermost layer stratum corneum [185]. However, studies have evidenced the improved skin permeability of AgNPs via coating/functionalizing with various polymers and peptides, which supports their use in skin cancer therapy.

In addition, the AgNPs can be conjugated with many other anticancer agents, including other metallic NPs, to manifest synergistic activity. One such study by Ruiz and colleagues investigated the effect of Ag and platinum (Pt) conjugated NPs on human melanoma cell line (A375) [186]. The particle size of the Ag-Pt NPs was found to be 42 ± 11 nm, along with the zeta potential -30 mV. Further, the IC_50_ value of Ag-Pt NPs was determined to be 50 µg/ml after incubation for 5 days. However, no cytotoxicity was observed in normal fibroblast cell line at the concentration range of 10–50 µg/ml. These results encourage them to participate further in preclinical studies.

In order to overcome the drawbacks associated with chemical routes of AgNPs synthesis, many researchers employed biogenic synthesis methods. The most widely exploited components in biogenic synthesis are plants, fruits, peels, and seeds-based extracts [35]. However, this could afflict the natural and food resources at the global level leading to impairing environmental sustainability. Therefore, a recent study by Himalini and team synthesized the AgNPs using extracellular fungal extract of *Fusarium incarnatum* to treat skin melanoma [187]. The fungus secretes a wide range of proteins and enzymes that can be taken part as capping and reducing agents in AgNPs formation. With a particle size of 10 nm, the synthesized AgNPs rendered maximum cytotoxicity in human skin melanoma cell line (SK-MEL-3) with an IC_50_ value of 17.70 µg/ml. The in vitro results exhibited promising results. However, further investigations on their biocompatibility and biodistribution in preclinical settings are much needed to confirm their safety profile.

An interesting study by Capanema and co-workers investigated the synergistic antimelanoma activity of AgNPs and DOX [188]. The authors synthesized the AgNPs in a green route using carboxymethylcellulose (CMC) as a capping agent. Further, they conjugated the DOX in to the crosslinked network of CMC. Finally, citric acid was attached (CA) to yield stable nano colloids of Ag with 10 nm diameter. The resulting nanosystem yielded maximum cytotoxicity in human melanoma cell line (A375) than normal human embryonic kidney cell line (HEK-293-T), making them suitable systems for melanoma therapy.

So far, we have found many studies exploring the synergistic/combinatorial activity of AgNPs and chemotherapeutic agents or other metallic NPs against skin cancer. Here is a study by Kuang and colleagues that explored the synergistic activity of AgNPs and immunotherapy against melanoma conditions [189]. In this study, the authors have synthesized the sucrose-coated AgNPs to enhance their stability for a more extended period. TEM analysis displayed that the particle size of plain AgNPs was 2.3 ± 0.4 nm; however, it has been shifted to 6.7 ± 3.2 nm, followed by sucrose coating. Thereafter, the combination of S-AgNPs and anti-PD-1 was tested on melanoma-bearing C57BL/6 mice. The in vivo results displayed more significant tumor growth inhibition in the mice group treated with S-AgNPs and anti-PD-1 compared to free anti-PD-1. Further, the ability of S-AgNPs in upregulating tumor PD-L1 was proved by the results of quantitative real-time PCR conducted on isolated tumors after S-AgNPs treatment alone. Based on this evidence, the small-sized S-AgNPs could be considered a potential adjuvant for immunotherapy.

It has been widely reported that the anticancer activity of AgNPs highly depends on their size. For instance, the smaller the size greater the transportation, tumor accumulation, and cellular uptake. However, plenty of controversies are still going on related to the size-mediated uptake of AgNPs. In this regard, Wu and co-workers investigated the variation in cellular uptake of different sized AgNPs using murine melanoma cell line (B16F10) [190]. AgNPs with 100 nm particle size displayed maximum uptake efficiency than 20 nm AgNPs. Furthermore, the migration rate of 100 nm AgNPs through plasma membrane was deemed very low compared to 5 nm AgNPs. Nevertheless, pre-treatment using chlorpromazine hydrochloride (clathrin-based endocytosis inhibitor) declined the uptake of all sized AgNPs (5, 20, 50, and 100 nm). Also, the internalization efficiencies of 5, 20, and 50 nm AgNPs were remarkably reduced due to methyl-β-CD (caveolin-mediated endocytosis inhibitor). Finally, 50 and 100 nm AgNPs uptake were low due to the 5-(N-ethyl-N-isopropyl) amiloride (macro-pinocytosis inhibitor). All these results suggest that the size of AgNPs is not only a factor that affects the efficiency of uptake in melanoma cells but also the type of endocytosis that is held responsible for the uptake mechanism. Overall, the clathrin-based endocytosis might be contemplated as a typical pathway for AgNPs uptake in melanoma cells.

Another exciting investigation by Netchareonsirisuk and team explored the role of different capping agents in AgNPs cytotoxicity using healthy (CCD-986SK) and cancer (A375) cell lines [191]. In this study, the authors synthesized AgNPs using sodium borohydride (NaBH_4_) as a reducing agent and alginate (natural) or poly (4-styrenesulfonic acid-comaleic acid) sodium salt (PSSMA) (synthetic) as a capping agent. The particle size of both the AgNPs ranged between 10.5 and 11.5 nm. Further, the zeta potential of alginate-AgNPs was found to be in the range of -31.3 to -36.0 mV, whereas PSSMA-AgNPs displayed -26.4 to -32.0 mV. However, both the zeta potential values indicated that the AgNPs were in stable form due to suffice repulsion between particles impeding aggregation. Finally, coming to the main part of the study, i.e., cytotoxicity in cell lines. The results revealed that alginate-AgNPs (natural capping agent) were highly toxic to cancer cells than normal skin cells. However, unaltered AgNO_3_ and PSSMA-AgNPs (synthetic capping agent) exhibited significant toxicity to both normal and melanoma cell lines. Further, the IC_50_ values ranged from 26–46 µg/ml for alginate and PSSMA-based AgNPs in melanoma cells. Overall, it can be concluded that the cancer cells (A375) were more sensitive to AgNPs than normal cells (CCD-986SK), making them eminent candidates for skin cancer therapy.

AgNPs/ *Penicillium citrinum* CGJ-C2/ squamous cell carcinoma [192]**,** AgNPs/ *Annona muricata P*/ melanoma [193]**,** AgNPs/ *Trapa natans*/ squamous cell carcinoma [194]**,** AgNPs/ *Rubia cordifolia L*/ melanoma [195]**,** AgNPs, 5-aminolevulinic acid/ *Bacillus licheniformis*/ melanoma, squamous cell carcinoma [196]**,** AgNPs, sodium dichloroacetate/ melanoma [197]**,** Au-AgNPs/ starch/ melanoma [198]**,** miR-148b, AgNPs/ squamous cell carcinoma [199]**,** AgNPs/ bovine serum albumin/ melanoma [200]**,** AgNPs/ Indigofera hirsuta L/ melanoma [201]**,** betulin, AuNPs/ polyethylene glycol/ melanoma [202] are few more recent investigations that were conducted for the treatment of skin cancer condition.

### Cerium oxide nanoparticles

Cerium oxide NPs (CeO_2_ NPs) are unique kind of metal oxides that possess both redox regulation ability and enzyme-like activity. They have shown promising results in many biomedical applications, including cancer therapy. The enzyme mimetic activity of CeO_2_ NPs such as superoxide dismutases (SOD), catalase (CAT), photolyase, deoxyribonuclease I (DNase I), oxidase, and peroxidase furnish them with the ability to modulate the ROS levels. Cerium consists of two different oxidation states such as Ce^3+^ (reduced) and Ce^4+^ (oxidized), due to which they act as an oxidant in cancer cells (produces ROS in acidic pH) and antioxidant in healthy cells (scavenges ROS in neutral pH). They display ROS scavenging activity due to their self-regeneration cycle of Ce^3+^/Ce^4+^ and oxygen vacancy on the cerium oxide surface [203]. Multiple studies have been conducted to explore the mechanism behind the anticancer activity. For instance, a study reported that CeO_2_ NPs increased the ROS production in tumor cells leading to DNA fragmentation and further caused apoptosis through mitochondrion-mediated apoptosis signaling pathway (confirmed by cytochrome c release, activated caspase-3, and caspase-9) [204]. Another study revealed that CeO_2_ NPs inhibits the formation of myofibroblasts (a primary unit of cancer progression) in tumor cells resulting in termination of tumor invasion. Utilizing this advantage, a study by Aplak and team investigated the antimelanoma potential of CeO_2_ NPs in a human melanoma cell line (A375) [205]. In this study, the authors purchased the commercially available water-dispersed CeO_2_ NPs with a mean diameter of 1–10 nm after stabilizing them using sodium polyacrylate. Corresponding to the previous studies, the CeO_2_ NPs induced mitochondrial dysfunction even in melanoma cell lines due to their SOD-mimetic activity (elevated ROS production), finally yielding cell death. An interesting study by Ali and co-workers reported that the commercially purchased CeO_2_ NPs with a particle size of 25 nm induced significant cell death in a human melanoma cell line (A375) via DNA damage (measured via comet assay) [206]. Another study by Pešic and colleagues revealed that the synthesized CeO_2_ NPs (4 nm particle size) were more toxic to melanoma cells (518A2) with an IC_50_ value of 125 µM compared to normal cells (keratinocytes HaCaT and lung fetal fibroblasts MRC-5) [207]. All these evidences encourage the CeO_2_ NPs to be a promising candidate for the treatment of skin cancer conditions, especially melanoma. However, further safety and therapeutic efficacy studies in the animal model could strengthen the obtained in vitro results.

### Miscellaneous inorganic NPs

In previous sections, we discussed different inorganic NPs frequently taken part in skin cancer therapy. However, there are still more inorganic NPs yet to be explored meticulously for their anti-skin cancer properties. For instance, bioactive glass NPs [208], terbium oxide NPs [209], graphene oxide NPs [210], and so on [211] are some of the potential inorganic NPs that exhibit significant anticancer activity. However, no studies were notably reported on their anti-skin cancer properties. This shows that there is a huge research gap, where many biomedical researchers can explore the potential of the aforementioned inorganic NPs for the treatment of skin cancer.

Apart from those unexplored inorganic NPs, a few more metallic NPs have shown promising results in skin cancer therapy. But many more studies are still required to support their current stature in treating skin cancer conditions. One such metallic nanomaterial is platinum NPs (Pt-NPs). The Pt-NPs are made out of a noble metal that possess unique physicochemical properties, including surface plasmon resonance (helps in PDT). Reports suggest that Pt-NPs can cause DNA strand breakage in the soluble form [212, 213]. On the other arrow, plenty of platinum-based chemotherapeutic agents (oxaliplatin, carboplatin, cis-platin, and phenanthriplatin) are already being used in cancer therapy. Owing to this supremacy, a recent study by Mukherjee and team investigated the combinatorial/synergistic antimelanoma potential of DOX conjugated Pt-NPs in both in vitro and in vivo settings [214]. The particle size of DOX@Pt-NPs analyzed by TEM micrographs displayed 5–20 nm. In contrast, DLS studies exhibited 50–70 nm particle size for plain Pt-NPs, which further increased to 220 ± 8.5 nm after conjugating with DOX. The biocompatibility study in four normal cell lines (HUVEC, NIH-3T3, ECV-304, and EA. hy926) exhibited more than 90% cell viability for Pt-NPs at 20 µM concentration (24 h incubation). Subsequently, the free DOX exhibited an IC_50_ value of 2.5 µM, whereas DOX@Pt-NPs displayed only 1.25 µM indicating the developed nanosystem is highly effective in cancer cells than normal cells. Lastly, tumor growth inhibition was higher in the melanoma-bearing C57BL6/J mice group treated with DOX@Pt-NPs than in free DOX and free Pt-NPs treated groups, proving the combinatorial/synergistic treatment approach is a suitable strategy for melanoma therapy.

Copper is another metal that has proved its stance as an anticancer agent in nanoform. However, there are limited investigations on the anti-skin cancer properties of copper NPs (CuNPs). Among those few studies, a research team led by Mita Chatterjee Debnath at CSIR-Indian Institute of Chemical Biology, India, have reported the effect of CuNPs synthesized using floral extract of plant *Quisqualis Indica Linn* on melanoma condition in both in vitro and in vivo set-up [215]. The SEM analysis showed that the average particle of CuNPs was 39.3 ± 5.45 nm. The developed CuNPs display more than 80% cell viability in normal fibroblast cell line (NIH-3T3) (concentration range of 40–120 µg/ml). Contrarily IC_50_ value of CuNPs in mouse melanoma cell line (B16F10) was found to be 102 µg/ml. Based on ROS and GSH estimation, oxidative stress was found to be the reason behind melanoma cell death. Further, significant inhibition of tumor growth was witnessed in melanoma-bearing BALB/c mice treated with CuNPs than plain floral extract of plant *Quisqualis Indica Linn*, making CuNPs an excellent agent in melanoma therapy.

Some of the recent research findings on inorganic NPs-mediated treatment strategies for skin cancer therapy are depicted in Table 2.

**Table 2:** Latest investigations on inorganic NPs-based therapeutic approaches for skin cancer

**Type**	**Therapeutic agent**	**Particle size**	**In vitro cytotoxicity study**	**Animal model**	**Route of administration**	**Ref**
Mesoporous silica nanoparticles	Stimulator of interferon genes (STING)	80 nm (particle size)/ 5–10 nm (pore size)	Mice melanoma cell line (B16F10)	Melanoma-bearing C57BL/6 mice	Intratumoral	[216]
Mesoporous silica nanoparticles	JQ-1 (immunotherapy)/ Polydopamine (PTT)	174.0 ± 2.4 nm	Mice melanoma cell line (B16F10)	Melanoma-bearing male C57BL/6 mice	Intratumoral	[217]
Mesoporous silica nanoparticles	Axitinib/ sgPD-L1/ CRISPR/Cas9	135 ± 8.7 nm	Mice melanoma cell line (B16F10)	Melanoma-bearing female C57BL/6 mice	Intravenous	[218]
Carbon nanotubes	Multiwalled carbon nanotubes (PTT)	3–15 nm (walls)/ 5–20 nm (outer diameter)/ 1–10 µm (length)	Mice melanoma cell line (B16F10)	Melanoma-bearing female C57BL/6 J mice	Intravenous	[219]
Carbon nanotubes	Multiwalled carbon nanotubes (Nanocyl™ NC3100)	9.5 nm (diameter)/ 1.5 µm (length)	Mice melanoma cell line (B16F10)	Mice	Intratumoral	[220]
Carbon nanotubes	Cytosine-phosphate-guanine oligodeoxynucleotide/ Anti-CD40 Ig/ Ovalbumin	20–30 nm (diameter)/ 0.5–2 µm (length)	-	Melanoma-bearing C57BL/6 mice	Intravenous	[221]
Zinc oxide nanoparticles	Zinc oxide nanoparticles	154.41–172.89 nm	Human epidermoid carcinoma cell line (A431)/ Human keratinocyte cell line (HaCaT)	-	-	[222]
Zinc oxide nanoparticles	Zinc oxide nanoparticles	10–20 nm	Human melanoma cell line (A375)	-	-	[223]
Zinc oxide nanoparticles	Zinc oxide nanoparticles	50 nm	Human epidermoid carcinoma cell line (A431)	-	-	[224]
Gold nanoparticles	Gold nanoparticles/ HuAL1 and C7H2 peptides	270 ± 22 nm	Mice melanoma cell line (B16F10)/ Human foreskin fibroblast cell line (Hs68)	Melanoma-bearing C57BL/6 mice	-	[225]
Gold nanoparticles	Gold nanoparticles (PTT)	157 ± 5 nm	Mice melanoma cell line (B16F10)	Melanoma-bearing severe combined immunodeficient (SCID) hairless mice (Xenograft-A375)	Intratumoral	[226]
Gold nanoparticles	Gold-iron oxide nanoparticles	37.8 nm	-	Melanoma-bearing C57BL/6 mice	Intravenous	[227]
Gold nanoparticles	Cytosine-guanine oligodeoxynucleotide/ CSIINFEKL (peptide-based tumor antigen vaccine)	146.30 ± 1.93 nm	-	Melanoma-bearing female C57BL/6 mice	Intravenous	[228]
Silver nanoparticles	Silver nanoparticles (PTT)	100 nm	Mice melanoma cell line (B16F10)	Melanoma-bearing athymic nude mice	Topical	[229]
Silver nanoparticles	Silver nanoparticles	35 ± 15 nm	Mice melanoma cell line (B16F10)	Melanoma-bearing male C57BL/6 J mice	Subcutaneous	[230]
Silver nanoparticles	Silver nanoparticles/ *Salmonella*	15 nm	Mice melanoma cell line (B16F10)	Melanoma-bearing BALB/c mice	Intravenous	[231]
Silver nanoparticles	Silver nanoparticles/ Indocyanine green (PTT)	131.5 ± 2.7 nm	Mice melanoma cell line (B16F10)	Melanoma-bearing athymic nude mice	Intravenous	[232]
Cerium oxide nanoparticles	Cerium oxide nanoparticles	5 nm	Human melanoma cell line (A375)	Melanoma-bearing athymic nude mice (Xenograft-A375)	Intraperitoneal	[233]
Platinum nanoparticles	Iron-platinum nanoparticles/ 5-Fluorouracil	6 ± 1 nm	Human skin fibroblasts cell line/ Human basal-cell carcinoma cell line	-	Topical	[234]
Platinum nanoparticles	Platinum nanoparticles (PTT)	12.2 ± 0.7 nm	Mice melanoma cell line (B16F10)	-	-	[235]
Copper nanoparticles	Copper nanoparticles	60–80 nm	Human melanoma cell line (A375)	-	-	[236]
Titanium dioxide nanoparticles	Titanium dioxide nanoparticles (PDT)	20–90 nm	Mice melanoma cell line (B16F10)	-	-	[237]
Titanium dioxide nanoparticles	Titanium dioxide nanoparticles/ Gold nanoclusters/ Graphene (PDT)	206–384 nm	Mice melanoma cell line (B16F10)	Melanoma-bearing female BALB/c athymic nude mice	Intravenous/ Intratumoral	[238]

## Polymer-based nanoparticles for skin cancer therapy

Polymer-based NPs are specialized drug carriers developed from either synthetic or natural polymers with varying sizes of 10–1000 nm [37, 239–241]. The polymer-based NPs are largely segregated into two groups such as nanocapsules (cavities surrounded by polymeric shell/branch) and nanospheres (solid matrix system). Further, they are sub-categorized into different types, i.e., micelles, dendrimers, polymersomes, polyplexes, etc., based on their shape and polymer properties [87]. These NPs are capable of conjugating, adsorbing, entrapping, or encapsulating the anticancer agents (hydrophilic and lipophilic drugs, monoclonal antibodies, genes, etc.) for controlled release, tumor targeting (active/passive), protection in physiological conditions, and increased tumor uptake, which could substantially improve the cancer treatment [242, 243]. Thereafter, due to the simple preparation technique, biocompatibility, biodegradability, and less cost, many researchers showed exceptional interest in developing anticancer agents loaded with polymer-based NPs for treating skin cancer conditions. In the upcoming sections, we will be thoroughly discussing different types of polymer-based NPs, such as micelles, dendrimers, and polymeric NPs followed by their applications in skin cancer therapy.

### Polymeric micelles

Polymeric micelles (PMs) are self-assembled colloidal particles with a size range of 5–500 nm, generally made of amphiphilic di- or tri-block copolymers. At critical micellar concentration (CMC), the di-block copolymers such as polyethylene glycol (PEG) and polystyrene, graft copolymers like G-chitosan and stearic acid, and triblock copolymers such as polyethylene oxide rapidly self-assembles in aqueous medium to form hydrophobic core and hydrophilic shell, which is termed as PMs [244, 245]. However, there are witnesses of reverse PMs too, with a hydrophilic core and hydrophobic shell [246]. One more interesting part that makes the PMs a unique drug carrier is that the amphiphilic copolymers exert a relatively low CMC compared to low molecular weight surfactants. Thus, the PMs can remain stable even at very low polymer concentrations being insensitive to dilutions in physiological conditions [247]. The hydrophobic core of PMs greatly helps in encapsulating numerous lipophilic anticancer agents, while the hydrophilic shell furnishes a stealth feature to the PMs. The stealth feature denies the PMs entry into RES, thereby prolonging their availability in the systemic circulation, making them available at the tumor site. Nevertheless, their small particle size supports excellent tumor uptake through the leaky vasculature compared to other drug carriers. Owing to these excellent specifications, there is a tremendous surge in the development of micelles for skin cancer therapy.

**Fig. 7:**
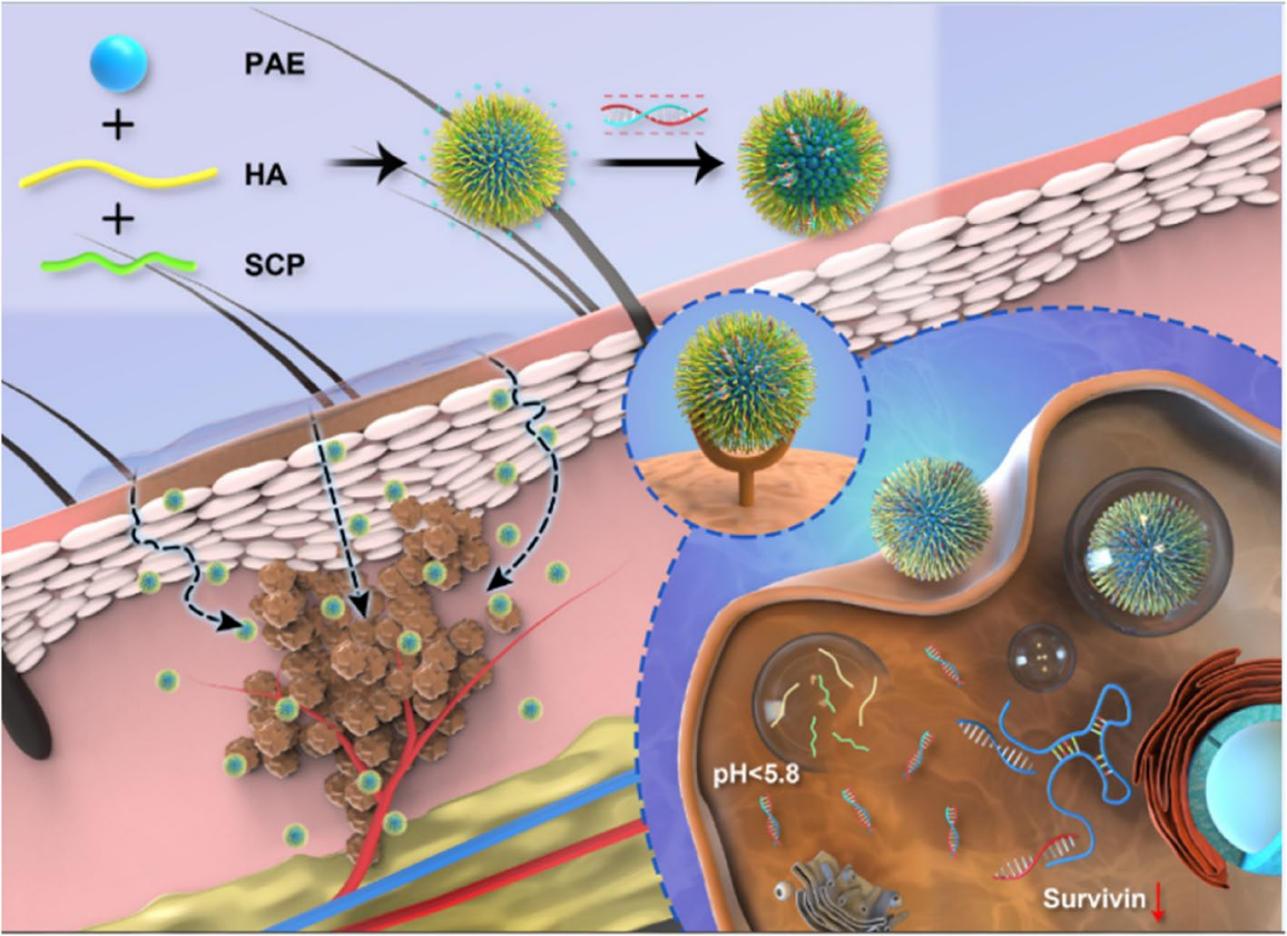
Schematic illustration of the design and therapeutic strategy of SHP. Part I: Synthesis of PAE and preparation of SHP micelle from the PAE, HA and SCP. Part II: Topical application of SHP/SiRNA induces survivin slicing in skin melanoma. (1) SHP/SiRNA nanocomplexes penetrate through the skin stratum corneum and target to melanoma locates at the interface of epidermis and dermis. (2) SHP/SiRNA-survivin nanocomplexes are uptaken by melanoma cells. (3) SHP/SiRNAsurvivin nanocomplexes escaped from the lysosome, release the siRNA that bind to the targeting RNA, followed by slicing survivin, which possesses the great potential to induce the significant apoptosis to melanoma cells in vitro and retard the melanoma progression in vivo, reproduced with permission from [249], copyright 2020, Elsevier

Recently, an investigation by Xu and team reported the development of PTX-loaded PMs to treat cutaneous melanoma via a topical route [248]. In this study, the authors first developed ibuprofen-modified methoxy PEG-PEI-based micelles loaded with PTX. Next, PTX@PMs were incorporated into Carbopol 940 hydrogel to improve the skin residence time. The DLS analysis exhibited a particle size of 221.7 ± 4.76 nm, a zeta potential of 20.7 ± 0.5 mV, and a 91.98% loading capacity for PTX@PMs. Further, based on FT-IR study, it has been found that the micelle formulation disorganized the lipid and keratin structure in the skin, thereby elevating the fluidity of lipidic compounds in the skin’s first layer. The positive charge of PMs confirmed by the zeta potential study enhanced the uptake in murine melanoma cells yielding maximum cell death compared to free PTX (Taxol®). However, in melanoma-bearing Kunming mice, PTX@PMs gel formulation + free PTX (Taxol®) exhibited more significant tumor growth inhibition compared to PTX and PTX@PMs gel formulation alone. In another investigation by Wang and colleagues, a cationic polymer (SCP-HA-PAE) was designed by attaching the hyaluronic acid (HA) and skin cell-penetrating peptide (SCP) to the amphipathic polymer (poly β-amino esters, PAE) [249]. Next, the authors used this polymer (SCP-HA-PAE) to develop pH-switchable siRNA@PMs (siRNA@SHP) for treating cutaneous melanoma via a topical route (Fig. 7). With a particle size of 170 nm, the developed siRNA@SHP displayed highest antimelanoma activity compared to free siRNA in both in vitro and in vivo setup. For topical melanoma therapy, another research team recently developed a nucleotide cyclase inhibitor MDL-12,330A20 loaded polypept(o)ide micelles [250]. The particle size was reported to be 76 nm for MDL@PMs; surprisingly, the plain PMs exhibited 98 nm, per DLS analysis. Further, it has been found that MDL@PMs suppress the cAMP formation in tumor tissue and melanoma growth more efficiently than free MDL. All these results demonstrate that PMs are highly efficient nanocarriers for delivering both small and large molecules via a topical route for improved melanoma therapy.

Although PTT is an outstanding treatment approach for superficial tumors, including melanoma, suitable photothermal agents with strong light absorbance, decent photostability, high photothermal conversion efficiency, and biocompatibility are needed to perform this therapy without fail and incompetence. Among many photothermal agents, aggregation-induced emission luminogens (AIEgen) have gained significant interest due to their excellent photobleaching resistance properties [251]. However, they face solubility issues, due to which they are administered via the intravenous route. The problem did not end there; further, it takes a minimum of 12 to 24 h for them to accumulate on the tumors due to their long systemic circulation period. This process is highly tedious for skin cancer treatment. Thus, to overcome these issues, Wei and co-workers developed the AIEgen (NIR950) loaded pH-sensitive polymer, i.e., methyl ether poly(ethylene glycol)-poly(β-aminoester) (mPEG-PAE) based PMs and then concentrated them on the tips of dissolving microneedles (MNs) for efficient PTT against cutaneous melanoma [252]. The developed PMs were found to be in size range of 80 to 125 nm, depending upon the varied concentration of loaded NIR950. With the help of dissolving MNs, NIR950@PMs rapidly accumulated at the skin melanoma site. Further, in an animal model, significant tumor inhibition was observed with only single administration of NIR950@PMs@MNs and one-time laser irradiation, proving the impact of PMs and MNs in efficient PTT against cutaneous melanoma. Nonetheless, there are a wide range of stimuli-responsive MNs (both internal and external) that could actively take part in skin cancer therapy with much more efficiency than conventional MNs or NIR triggered MNs [253]. These results further indicate significant potential for clinical superficial melanoma therapy.

### Dendrimers

Dendrimers are hyperbranched polymeric macromolecules with well-defined sizes and shapes consisting of innumerable branching from a central core, becoming a tree-like structure. The branched layers in dendrimers are termed “generations” (G) [254–256]. The first discovery of dendrimers was in 1978 by organic chemist Fritz Vogtle. Dendrimers are frequently synthesized by either convergent or divergent methods. In a converged method, the dendrimer grows inward, beginning with end groups, whereas, in a divergent method, the dendrimer grows outward from a functional core molecule in a stepwise manner [257]. Some of their common types are poly(amidoamine) (PAMAM) dendrimers [258], PEGylated dendrimers [259], polyether-copolyester dendrimers [260], poly(propylene imine) dendrimers [255], peptide dendrimers [261], etc. Although the dendrimers are classified under polymer-based NPs, they possess a unique structure comprised of three major components such as (1) central core, (2) repetitive branching units, and (3) terminal groups. The increase in the number of repeated branching units regulates the generations of the dendrimers, which has a direct relation with dendrimer size and globular shape (higher the generation, larger the particle size). Further, the terminal groups of dendrimers are responsible for modifiable surface functionalities (common terminal functional groups are COOH, COONa, NH_2_, and OH). These groups specifically enable dendrimers to conjugate with various tumor-targeting moieties [262, 263]. Nevertheless, all these characteristic features allow dendrimers to either conjugate/encapsulate the anticancer agents in the core or on the surface, devising them as an interesting drug carrier for skin cancer therapy without many side effects.

The hedgehog signaling (Hh) pathway is primarily active in many cancer conditions, including skin cancer, which enacts a critical job in cancer cell multiplication, differentiation, and survival [264]. Thus, the therapeutic agents that potently inhibit the Hh pathway provide an efficient treatment opportunity against cancer. In the year 2012, FDA approved a potential Hh inhibitor named Vismodegib (VDG), a small molecule with a molecular weight of 421.3 g/mol for oral administration (150 mg/day) in patients with a denial of surgery or radiotherapy [265]. However, their therapeutic ability is hindered due to poor aqueous solubility and side effects such as musculoskeletal spasms, alopecia, etc. In the quest to overcome these issues in skin cancer therapy (basal cell carcinoma; BCC), a research team came up with PAMAM-based dendrimers (D) loaded with VDG for site-specific delivery via topical route [266]. Further, they also studied the effect of two different types of PAMAM-D, such as 4.0 generation PAMAM-D with terminal primary amine (DG4.0) and 4.5 generation with carboxylic acid termination (DG4.5) skin permeability. It was found that VDG@DG4.0 showed better skin penetration in an ex vivo setup. Nevertheless, the developed VDG@PAMAM-D exhibited non-conventional fluorescence that allowed for monitoring of skin penetration resulting in the theragnostic potential of dendrimers. Another interesting investigation by Wang and colleague developed cRGD peptide attached pH and redox triggered G4 PAMAM dendrimers (D) loaded with DOX for targeted treatment of melanoma [267]. The developed DOX@RGD-D presented a particle size of 17.41 ± 0.36 nm. Further, in vitro cytotoxicity study in a murine melanoma cell line (B16F10) displayed maximum cell death compared to free DOX and DOX@D. Also, the IC50 value DOX@RGD-D was approximately 2–6 folds lower compared to DOX@D. Lastly, the cellular uptake mechanism revealed that DOX@RGD-D interacted with the plasma membrane of melanoma cells via specific identification of RGD peptide with integrin ανβ3 and was eventually internalized via clathrin- and caveolin-mediated endocytosis. Thus, it can be concluded that dendrimer-based nanocarriers could efficiently participate in skin cancer therapy.

PAMAM dendrimers are widely used in drug delivery applications. However, they lack traceability in their original form and also possess intrinsic cytotoxicity towards normal cells, which made them poor performer in clinical safety studies. Thus, a research team developed label-free fluorescent PAMAM dendrimers (D) via modifying their terminal groups using acetaldehyde, which produced strong green fluorescence due to the C = N bonds of the resulting Schiff Bases and also reduced their intrinsic cytotoxicity behavior [268]. Further, the fluorescent PAMAM-D displayed excellent intracellular tracking in melanoma cells (SK-MEL28) via PEGylation. It was also witnessed that PEGylated fluorescent PAMAM-D exhibited enhanced loading and controlled delivery of DOX compared to plain PAMAM-D. Finally, the developed dendrimers endowed maximum melanoma cell death compared to free DOX due to enhanced uptake of PEG-PAMAM-D into cancer cells followed by intracellular DOX release. This supports fluorescent PAMAM dendrimers to be an efficient nanocarrier for melanoma therapy with the additional opportunity of trackability. Another study by Smith and co-workers explored the efficiency of G5 PAMAM dendrimers and poly(d,l-lactic-co-glycolic acid) based nanoformulation as an adjuvant melanoma therapy with cancer vaccine [269]. The melanoma-bearing C57BL/6 J mice treated with adenovirus-based cancer vaccine (Ad5-TRP2) and PLGA-PAMAM-D nanoformulation exhibited significant enhancement in TAA-specific T cells in the peripheral blood with reduced tumor burden. Nevertheless, the cell-based pathways suggested that the adjuvant nanoformulation administration created an inflammatory environment at the tumor site, which further attracted the activated TAA-specific CD8 + T cells to the area of the tumor resulting in enhanced vaccine efficacy.

### Polymeric nanoparticles

**Fig. 8:**
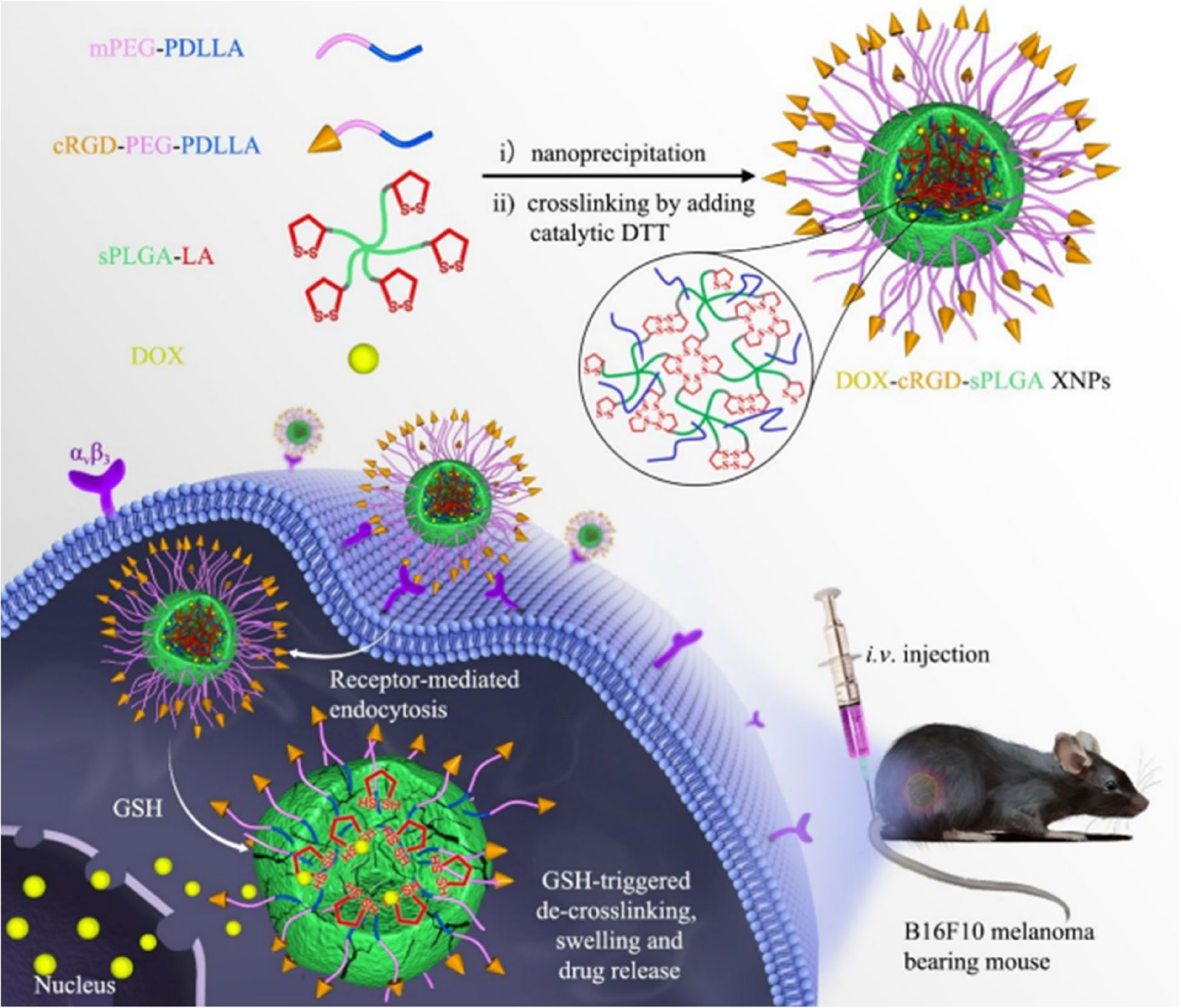
Illustration of cRGD-installed reduction-responsive crosslinked nanotherapeutics from star PLGA-lipoic acid conjugate (cRGD-sPLGA XNPs) for enhanced DOX delivery to B16F10 melanoma bearing mice in vivo, reproduced with permission from [277], copyright 2019, American Chemical Society

Polymeric NPs are simple and non-complex carrier systems that possess the ability to dissolve, disperse, encapsulate, or adsorb the anticancer agents for tumor targeting, sustained release, protection of therapeutic moieties, and so on [87]. Depending upon the method of preparation, the structure of NPs can vary from nanospheres (matrix system) to nanocapsules (reservoir system). In nanospheres, the therapeutic agents are simply dispersed throughout the particle–matrix system. In contrast, in nanocapsules, therapeutic agents are being held in an aqueous or oily cavity surrounded by a uni-polymeric membrane [94]. Further, the most commonly adopted biodegradable synthetic and natural polymers to develop this kind of NPs are poly(lactide-co-glycolide) (PLGA) [270], polylactide (PLA) [271]**,** polycaprolactone (PCL) [272], PLGA-polyethylene glycol (PEG) [273], alginate [274], gelatin [275], albumin [276], etc. Recently, an investigation by Wang and colleagues developed DOX-loaded cRGD-attached reduction-responsive crosslinked nanotherapeutics based on star PLGA-lipoic acid conjugate (cRGD-sPLGA XNPs) to achieve targeted delivery to melanoma (Fig. 8) [277]. With the particle size of 91 nm, the DOX@cRGD-sPLGA XNPs displayed maximum accumulation and significant cellular uptake in αvβ3 overexpressing murine melanoma cells (B16F10) followed by the efficient release of DOX into the nuclei compared to non-cRGD attached DOX@sPLGA XNPs, which released the DOX in the cytoplasm. Further, the IC_5O_ value of DOX@cRGD-sPLGA XNPs was found to be 0.92 µg/ml, which was 2 and 12.3 folds lesser than the non-targeted variant and Lipo-DOX (marketed PEGylated doxorubicin), respectively, indicating the active targeted PLGA-based nanosystem is a much-needed treatment approach for groundbreaking melanoma therapy.

Studies have displayed upregulation of CD44 receptors in human melanoma cells [278]. Therefore, CD44 receptor-specific targeting has gained much interest in developing a targeted drug delivery system to eradicate melanoma by enhancing the cellular uptake efficiency and tumor-specific distribution. In this regard, Chen and team came up with hyaluronan (HA, 49 kDa) attached cationic bovine serum albumin (BSA) NPs loaded with PTX for CD44 targeted melanoma therapy [279]. The biodistribution study in C57BL/6 mice melanoma lung metastasis model displayed maximum accumulation of PTX@HA-BSA NPs at the tumor site. Nonetheless, the IC_50_ value of PTX@HA-BSA NPs was found to be 12.96 ± 1.34 µg/ml. In contrast, commercial PTX product and non-targeted variant, i.e., PTX@BSA NPs endowed 19.04 ± 4.12 µg/ml and 28.34 ± 5.28 µg/ml, respectively, suggesting CD44 targeted chemotherapy is an efficient strategy to treat melanoma with low-cost HA as a targeting moiety.

So far, we have witnessed only chemotherapeutic agents deliverable active/passive targeted polymeric NPs. However, to manifest synergistic melanoma therapy, the development of a combinatorial system that can deliver chemotherapeutic agents and also exhibit excellent PTT was reported by Hao and co-workers [280]. In this study, the authors first designed a 5-FU and ICG (NIR dye for PTT) loaded monomethoxy-PEGPCL NPs (5-FU-ICG@MPEG-PCL NPs). Next, these NPs were loaded into HA-based dissolvable MNs (HAMNs) to yield 5-FU-ICG@MPEG-PCL@MNs. The MNs immensely helped to piece stratum corneum and deliver the NPs directly into the cutaneous melanoma region in a controlled manner upon NIR irradiation. This developed nano-micro system significantly inhibited the tumor growth compared to their individual modalities in melanoma-bearing BALB/c nude mice model, presenting the NIR-responsive 5-FU-ICG@MPEG-PCL@MNs as a noteworthy candidate to treat cutaneous melanoma without severe side effects caused by unnecessary system exposure of chemotherapeutics.

The latest research reports on skin cancer therapy via polymer NPs are enumerated in Table 3.

**Table 3:** Latest investigations on polymer NPs-based therapeutic approaches for skin cancer

**Type**	**Chief composition**	**Therapeutic agent**	**Particle size**	**In vitro cytotoxicity study**	**Animal model**	**Route of administration**	**Ref**
Polymeric micelles	D-α-tocopheryl succinates/ Chondroitin sulfate	Doxorubicin	137.87 ± 2.32 nm	Mice melanoma cell line (B16F10)	Melanoma-bearing C57BL/6 mice	Intravenous	[281]
Polymeric micelles	1,2-distearoyl-sn-glycero-3phosphoethanolamine-N-[maleimide-(polyethyleneglycol)-2000]	MCP-1 peptide/ KLAKLAK peptide	11.9 ± 2.3 nm	Mice melanoma cell line (B16F10)	Melanoma-bearing male C57BL/6 J mice	Intravenous	[282]
Polymeric micelles	Polyethylene glycol/ Hydroxydodecanoic acid	Doxorubicin	200 nm	Mice melanoma cell line (B16)	-	Intravenous	[283]
Polymeric micelles	D-α-tocopheryl-polyethylene-glycol-succinate	All-trans-retinoic acid	11.4 ± 0.1 nm	Human melanoma cell line (BRAFV600)	-	Topical	[284]
Polymeric micelles	D-α-tocopheryl polyethylene glycol 1000 succinate	Imiquimod	13.40 ± 0.16–14.90 ± 0.13 nm	-	-	Topical	[285]
Dendrimers	Poly(amidoamine)/ Poly (ethylene glycol)	Cytosine-phosphate-guanine oligonucleotides/ Doxorubicin	35 ± 4.2 nm	Mice melanoma cell line (B16F10)	Melanoma-bearing C57 mice	Intravenous	[286]
Dendrimers	Poly(amidoamine)	Cytosine-guanine dinucleotides	58–68 nm	Mice melanoma cell line (B16-OVA)	Melanoma-bearing female C57BL/6 mice	Intravenous	[287]
Dendrimers	Poly(amidoamine)/ Iron oxide	Doxorubicin	-	Mice melanoma cell line (B16F10)	Melanoma-bearing male C57BL/6 mice	Intravenous	[288]
Dendrimers	Poly(amidoamine)	Celecoxib/ Fmoc-L-Leucine	-	Squamous cell carcinoma (SSC-15)/ Human normal fibroblast cell line	-	-	[289]
Dendrimers	Poly(amidoamine)	5-Flurouracil	2.45 ± 0.06–3.75 ± 0.19 nm	Human melanoma cell line (A375)	-	-	[290]
Polymeric nanoparticle	Poly(lactic-co-glycolic acid)	Baicalin/ Hgp peptide fragment/ CpG fragments	123.6 nm	Mice melanoma cell line (B16F10)	Melanoma-bearing C57BL/6 mice	Intravenous	[291]
Polymeric nanoparticle	Poly(lactic-co-glycolic acid)	Baicalin/ Hgp peptide fragment/ CpG fragments	168.9 nm	Mice melanoma cell line (B16F10)	Melanoma-bearing female C57BL/6 mice	Intravenous	[292]
Polymeric nanoparticle	Poly(lactic-co-glycolic acid)/ D-α-tocopherol polyethylene glycol 1000 succinate	Paclitaxel/ PD98059 (MAPK inhibitor)	180 nm	Mice melanoma cell line (B16F10)	Melanoma-bearing nude mice	Intravenous	[293]
Polymeric nanoparticle	Bovine serum albumin	Curcumin	150 nm	Mice melanoma cell line (B16F10)	Melanoma-bearing male C57BL/6 J mice	Intraperitoneal	[294]
Polymeric nanoparticle	Chitosan/ Alginate	Doxorubicin	300 nm	Mice melanoma cell line (B16F10)	Melanoma-bearing female C57BL/6 mice	Intravenous	[295]

## Lipid-based nanoparticles for skin cancer therapy

Lipid-based NPs are distinctive carrier systems that contain either lipid monolayer (solid lipid nanoparticles and nanostructured lipid carriers) or lipid bilayer (liposomes, niosomes, ethosomes, etc.) along with solid lipid core (solid lipid nanoparticles), liquid lipid core (nanostructured lipid carriers), or aqueous core (liposomes, niosomes, ethosomes, etc.) in which the therapeutic agents are either dissolved or dispersed to deliver via several routes of administration [296, 297]. The anticancer agents that are hydrophilic in nature face the permeability issue, and hydrophobic anticancer moieties lack suffice aqueous solubility leading to poor therapeutic efficacy. In addition to that, chemotherapeutic agents have the tendency to destroy both normal and cancer cells when freely present in the physiological system. Nevertheless, specific anticancer agents are prone to degradation in either physiological or external environments (light, temperature, and humidity), deactivating their therapeutic properties. In order to overcome all these drawbacks, the lipid-based NPs are deemed the most appropriate carrier systems due to their unique lipidic composition, which was made from physiologic and/or biodegradable lipids [298]. The supremacy of lipid-based NPs includes controlled release, burdenless and easy formulation, compatibility, high drug loading efficiency (both hydrophilic and hydrophobic), and last but not least, lipid-based NPs are the highest number of nanomedicines that the FDA has approved so far (due to their safety profile) [299]. Some lipid-based NPs that have shown remarkable results in skin cancer therapy are solid lipid nanoparticles (SLNs), nanostructured lipid carriers (NLCs), liposomes, niosomes, transferosomes, ethosomes, and so on, which will be discussed thoroughly in the upcoming sections.

### Solid lipid nanoparticles

SLNs were first introduced in the year 1991 as colloidal lipid carriers with a typical size range of 50–1000 nm [300]. They were made of natural lipids such as fatty acids, steroids, waxes, monoglycerides, diglycerides, and triglycerides, which remain in a solid form at both ambient and physiological temperatures. As the name indicates, the core lipid matrix in SLNs is constituted by a solid lipid (0.1–30% w/w) that encapsulates either lipophilic or hydrophilic drugs depending upon the method of preparation followed by stabilization of the core lipid matrix using surfactants (0.5–5% w/w) [301]. Nevertheless, their ability to encapsulate anticancer agents and safely guide them to the tumor site to achieve controlled release without involving any permeability and toxicity issues has made them the most competing drug carriers for skin cancer therapy.

Concerning all these advantages, a study by Valdes and team developed 4-(N)-docosahexaenoyl 2′, 2′-difluorodeoxycytidine (DHA-dFdC) encapsulated SLNs to enhance the antimelanoma efficacy of DHA-dFdC via oral administration [302]. In vivo pharmacokinetics study displayed maximum oral bioavailability for DHA-dFdC@SLNs compared to free DHA-dFdC. This was further confirmed in the melanoma-bearing mice model, which exhibited a maximum survival rate for orally administered DHA-dFdC@SLNs than free DHA-dFdC. In another investigation, Kim and co-workers made an attempt to overcome the toxicity of intravenously administered docetaxel (DTX) by encapsulating it in SLNs to achieve sustained delivery for 24 h after oral administration [303]. Further, the DTX-loaded cationic SLNs were coated with glycocholic acid conjugated anionic polymer (D-SLN-CSG) to ensure they actively absorb through the distal ileum (via interactions with the apical sodium bile acid transporter) after oral administration. The in vivo study using C57BL/6 mice bearing melanoma displayed maximum inhibition of tumor after oral administration of D-SLN-CSG than intravenous administration of free DTX, supporting the use of SLNs-based DTX via oral route for enhanced antimelanoma efficacy.

Studies have proved that PTX is a more efficacious chemotherapeutic agent against melanoma than FDA-approved DTIC, also called the gold standard for melanoma therapy. This is because DTIC does not induce cell-surface exposure of calreticulin, a chief biomarker for immunogenic cell death. However, this is not the case with PTX. However, poor aqueous solubility and severe toxicity issues limit their potential application in melanoma therapy. Thus, Banerjee et al. developed robust PTX@SLNs attached with Tyr-3-octreotide (PST) for active targeted delivery to melanoma sites [304]. The results revealed PST exerted more apoptotic and anti-invasive effects in the murine melanoma cell line (B16F10) than DTIC. Further, the melanoma-bearing mice treated with PST showed the highest number of CD8 + T cells in their tumor region; due to this, PST exerted maximum inhibition of tumor growth than DTIC. Nevertheless, the PST potentially reduced the number of nodule formations in the lung metastasis model without any severe side effects, making them promising candidates for melanoma therapy.

Immunotherapy, targeted therapy, and chemotherapy are well known for skin cancer treatment despite their cost and side effects. In the quest to overcome the issues related to cost and side effects, scientists explored numerous phytoconstituents (CUR, RVT, quercetin, coumarin, etc.) with potential anticancer properties [305]. However, their poor physicochemical properties hinder their potential application in melanoma therapy. To overcome this circumstance, Palliyage and co-workers came up with CUR and RVT-loaded SLNs as a topical delivery system to treat aggressive melanoma conditions [306]. With an average particle size of 180.2 ± 7.7 nm, the negatively charged CUR-RVT@SLNs exhibited maximum cytotoxicity in the melanoma cell line (SK-MEL-28) with improved skin permeability (in vitro study using Franz diffusion cell). On the other arrow, an interesting study by Valizadeh and colleagues explored the potential antimelanoma property of *Zataria multiflora*’s essential oil-loaded SLNs [307]. With a particle size of 176 ± 8 nm and entrapment efficiency of 67 ± 5%, these SLNs endowed less than 13% melanoma cell (A-375) viability at 75 µg/ml concentration. In contrast, plain essential oil exerted more than 50% cell viability. All these reports strongly witness the potential of SLNs as eminent drug carriers in skin cancer therapy.

Some of the notable investigations on SLNs that endowed extraordinary results against skin cancer include omega-3 α-linolenicacid/ α-tocopheryl linolenate, sodium taurocholate, tween 20/ melanoma [308]**,** *Mentha longifolia*, *Mentha pulegium* essential oils/ stearic acid, span 60, tween 80/ melanoma [309]**,** octyl gallate/ Astrocaryum murumuru (seed butter), tween 80/ melanoma [310]**,** doxorubicin/ lecithin, sodium taurodeoxycholate/ melanoma, squamous cell carcinoma [311]**,** paclitaxel/ stearic acid, egg lecithin/ squamous cell carcinoma [312]**,** microRNA-34a, paclitaxel/ glyceryl monostearate, cholesterol, soy phosphatidylcholine, dimethyldioctadecyl ammonium bromide/ melanoma [313]**.**

### Nanostructured lipid carriers

NLCs are second-generation lipid NPs, which can also be considered an upgraded version of SLNs. Unlike SLNs, the NLCs are comprised of both solid lipids (fat) and liquid lipids (oil) in a ratio of 70:30 up to a ratio of 99.9:0.1 along with surfactants (1.5–5% w/v) [314]. The utilization of liquid lipids in NLCs helps overcome the drawbacks of SLNs, like low therapeutics loading and poor storage stability (expulsion of drug) by circumventing lipid crystallization. Some commonly used liquid lipids in the construction of NLCs are ethyl oleate, isopropyl myristate, glyceryl dioleate, and glyceryl tricaprylate. Further, studies have displayed that the appropriate selection of solid lipids, liquid lipids, and surfactants, along with their concentration, have a direct impact on particle size, drug loading capacity, controlled release ability, permeability, toxicity, and long-term stability of NLCs [315, 316]. Owing to all these advantages, the scientific community is anticipating their application in skin cancer treatment.

Recently, many studies have explored the adjuvant anticancer activity of local anesthetics like lidocaine, ropivacaine, bupivacaine, etc. It has been reported that lidocaine (LDC) can inhibit the growth of cancer cells via regulation of ABC transporters, promotion of pro-apoptotic pathways, regulation of epigenetic changes, preventing metastasis and angiogenesis [317]. Considering this as basement, a recent study by Moura and team investigated the synergistic antimelanoma effect of DTX and LDC loaded NLCs via topical route [318]. In this work, the authors first loaded NLCs with DTX, followed by incorporating the DTX@NLCs into lidocaine containing xanthan-chitosan hydrogel. The particle size of DTX@NLCs based on the DLS study was found to be 214.0 ± 10.9 with zeta potential and entrapment efficiency of -24.2 ± 0.3 and 97.3 ± 2.6%, respectively. Further, in vivo study on melanoma-bearing C57BL/6 J mice exhibited significant inhibition of tumor growth upon treatment with DTX@NLCs (intratumorally) + LDC@hydrogel (topically) compared to LDC-DTX@NLCs hydrogel (topically), DTX@NLCs hydrogel, and LDC @NLCs hydrogel. In addition to that, the reported DTX-loaded NLC formulation did not exhibit any side effects compared to free DTX. These results provide suffice hope to utilize NLCs as a potential drug carrier in the treatment of melanoma conditions.

The activation of STAT3 protein is commonly observed in many tumors, including melanoma, which plays a chief role in regulating tumor cell growth and survival, angiogenesis, and evasion of immune surveillance [319]. Therefore, scientists came up with a small molecule named Stattic to potentially inhibit the functionality of STAT3 via dimerization event [320]. Regardless of any other therapeutic agent, chemotherapy remains a gold standard for the treatment of melanoma. In this conspiracy, for the first time, Mohammadian and co-workers investigated the synergistic effect of DOX and Stattic in a murine melanoma cell line (B16F10) using NLCs as nanoplatform [321]. Here, the authors loaded Stattic into NLCs via a modified hot homogenization technique together with ultrasonication. Further, the in vitro cell line studies revealed maximum melanoma cell death upon treating with Stattic@NLCs + DOX compared to Stattic + DOX, Stattic, and DOX. Although the synergistic antimelanoma activity was witnessed using Sttatic and DOX, it was clearly identifiable that NLCs, as a carrier system, greatly enhanced the therapeutic efficacy by taming the drawbacks of free Stattic.

An interesting study by Imran and colleagues investigated the anti-skin cancer efficiency of RVT and quercetin (QUE) loaded NLCs via topical route [322]. The particle size of dual drug-loaded NLCs were found to be 191 ± 5 nm with a zeta potential -10.00 mV. Also, the developed NLCs exhibited 89% and 92% entrapment efficiency for RVT and QUE, respectively. Compared to conventional gel formulation (carbopol 934, 1.5% w/w), the NLC gel exhibited 3 folds higher deposition in skin layers. Nevertheless, the in vitro cytotoxicity study using a human epidermoid carcinoma cell line (A431) displayed an IC_50_ value of 86.50 µM for the NLC gel treated group, whereas conventional gel exhibited 123.64 µM. These results disclose that NLCs could potentially improvise the anticancer efficacy of RVT and QUE via topical route. Yet another study by the same research team studied the combinatorial effect of RVT and 5-fluorouracil (5-FU) loaded NLCs in the same skin cancer cell line (A431) [323]. Further, the results of MTT assay displayed an IC_50_ value of 22 μM for NLC gel and 52 μM for conventional gel. All these results immaculately suggest NLCs as a potential drug carrier that could create a road for promising skin cancer therapy via topical route.

### Liposomes, Niosomes, Transferosomes, and Ethosomes

Liposomes are vesicular drug delivery systems comprised of phospholipids and cholesterol to form a self-assembled lipid bilayer surrounding an aqueous core. The concept of liposome was first described by a British scientist “Bangham” in the early 1960s. Since then, liposomes have been widely involved in various biomedical applications as a potential drug carrier [324, 325]**.** Liposomes are categorized into 3 types such as small unilamellar vesicles (20–100 nm), large unilamellar vesicles (more than 100 nm), and multilamellar vesicles (more than 0.5 µm) based on their size and lamellarity. The most exciting part about liposomes is they seamlessly imbibe both hydrophilic (in central aqueous compartment) and hydrophobic (in outer lipidic bilayer) molecules at the same time [326]. Some commonly used phospholipids in liposome development are phosphatidyl serine, phosphatidyl inositol, phosphatidyl choline or lecithin, phosphatidyl glycerol, phosphatidyl ethanolamine or cephalin, and so on. These phospholipids rapidly undergo self-assembly in an aqueous environment to yield a lipidic bilayer (one or many) with a central aqueous compartment (one or many). Further, the cholesterol will be incorporated to uplift the stability of bilayers during their residence in biological fluids, thereby avoiding the premature release of therapeutics [327]. So far, many studies have elaborated on the ability of liposomes in anticancer therapeutics delivery.

No matter what technology we use today, there will always be an upgradation to it in the future. Similarly, to overcome certain drawbacks of liposomes related to permeability, physiological and storage stability along with cost, niosomes were introduced in 1970s by a cosmetic company L'Oréal. However, their first niosomal product was came into market in the year 1987. The only key difference between noisome and liposome is the usage of non-ionic surfactants (Spans, alkyl oxyethylenes, polysorbates, terpenoids, etc.) in the place of phospholipids to form a bilayer with an aqueous core [328, 329]. Although niosomes had proved their stance in addressing many drug delivery issues compared to liposomes, using non-ionic surfactant as a significant ingredient could cause potential toxicity issues. Thus, transferosomes were introduced by Cevc and Blume in the year 1992. Unlike liposomes and niosomes, the transferosomes are deformable or elastic vesicles comprised of phospholipids and edge activators (surfactants). The commonly used edge activators in transferosomes development are deoxycholate, dipotassium glycyrrhizinate, sodium cholate, Tweens, and Spans [330]. However, we already know that surfactants are prone to induce toxicity. Thus, to eliminate the usage of any surfactants, Touitou invented a novel vesicular structure named ethosomes. Ethosomes are formed by combining phospholipids, ethanol, and water to yield a lipid bilayer surrounding an aqueous-ethanolic core, where both hydrophilic and lipophilic drug can be encapsulated [331–333]. Nevertheless, all the four nano-carriers, such as liposomes, niosomes, transferosomes, and ethosomes were extensively studied for their anticancer therapeutics’ delivery ability to treat many cancer conditions, including skin cancer, without involving many side effects.

**Fig. 9:**
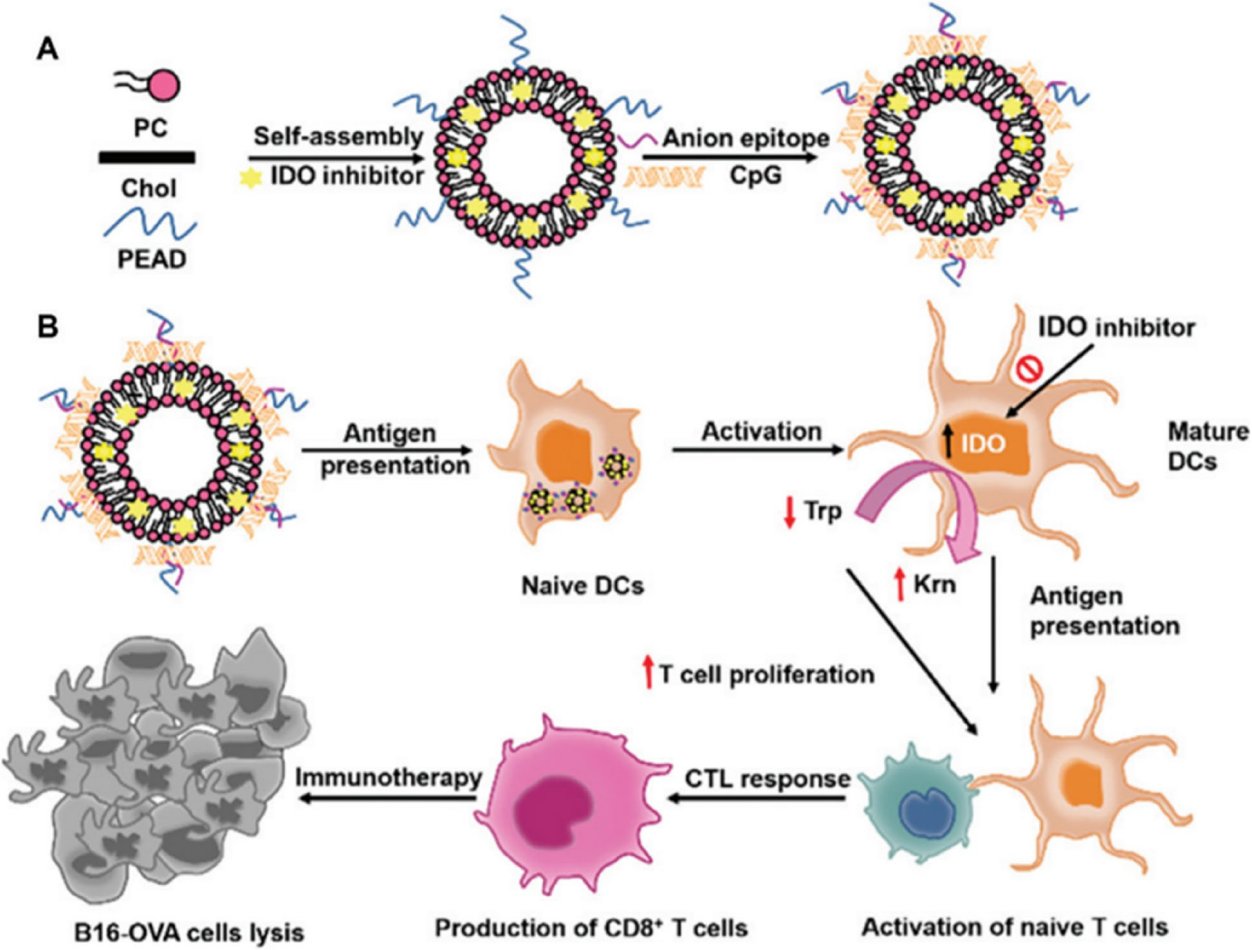
Schematic illustration of the formation of P/LNV loaded with tumor vaccines and IDO inhibitor (P/LNV@IDO/AE/CpG) and the action mechanism for immunotherapy (**A**) Preparation of P/LNV@IDO/AE/CpG. **B** Combination immunotherapy induced by P/LNV@IDO/AE/CpG. Naive dendritic cells (DCs) are activated and their maturation is induced by the antigens delivered by P/LNV@IDO/AE/CpG, which then present the processed peptide antigens to T cells, causing a strong cytotoxic T-lymphocyte (CTL) response. Tumor cells would be attacked by effector T cells. Besides, the presentation of 1-MT would inhibit the activity of IDO by decreasing the oxidization of tryptophan (Trp) to kynurenine (Krn), which further enhanced the antitumor immune response. Together, P/LNV@IDO/AE/CpG resulted in a superior combination immunotherapy against melanoma, reproduced with permission from [334], copyright 2021, Royal Society of Chemistry

A recent study by Su and colleagues reported the development of cationic liposomes loaded with peptide vaccine and indoleamine-2,3-dioxygenase (IDO) inhibitor for combinatorial melanoma therapy [334]. In this study, the authors first loaded 1-methyl-tryptophan (1-MT), an IDO inhibitor) into cationic liposomes. Further, they complexed (electrostatically) the liposomes with negatively charged epitopes (AE) that were derived from antigens of melanoma followed by conjugation of a strong TLR9 agonist, i.e., CpG, to yield a unique tumor vaccine (Fig. 9). Interestingly, the IDO inhibitor is a hydrophobic molecule, whereas peptide vaccine is a hydrophilic one. Regardless of that, the developed liposomal formulation efficiently encapsulated both the peptide vaccine and 1-MT (IDO inhibitor) in an aqueous core and lipid bilayer, respectively, and helped to enhance their presentation to DCs via efficient uptake, which effectually encouraged the cytotoxic T lymphocyte to eliminate melanoma cells. Overall, the liposomal formulation loaded with peptide vaccine and IDO inhibitor displayed a significant tumor inhibition than individual liposomal components, ensuring the lipo-based combinatorial immunotherapy provides a promising melanoma therapy platform.

As mentioned earlier in this section, to overcome the drawbacks of liposomes, such as poor stability and high production cost, Obeid and team developed a cationic niosomal formulation loaded with anti-luciferase siRNA to treat melanoma conditions [335]. The siRNA, in its free form, suffers from poor cell membrane permeability and stability. However, siRNA@niosomal formulation significantly suppressed the luciferase expression compared to free siRNA in both murine melanoma cell line (B16F10) and melanoma induced BALB/c nude mice (intratumoral administration). These results suggested that the niosomes are the potential drug carriers for siRNA delivery in melanoma therapy.

Rose Bengal (RB) is a synthetic dye commonly used in ophthalmology as a diagnostic tool. RB has recently gained significant attention due to its ability to act as a sono-photosensitizer, which can be employed in sono-PDT to kill cancer cells [336]. However, RB is a hydrophilic compound with a molecular weight of 1017.64 g/mol and anionic change in solution form. This characteristic feature of RB hinders its permeability through skin layers for treating cutaneous melanoma conditions. In order to surpass this barrier, Demartis and co-workers came up with an idea to develop transferosomal formulation loaded with RB to enhance their skin permeability that potentially suppresses the cutaneous melanoma lesion [337]. Here, the authors used a modified reverse-phase evaporation method to develop robust RB@transferosomes with a particle size of 206 nm, zeta potential of -45 mV, and 94% loading efficiency. The ex vivo study evidenced that RB@transferosomes significantly enhanced the permeation of RB compared to its free form (78.31% vs. 38.31%). Considering the outcome of the cytotoxicity studies, it can be concluded that transferosomes represent as a suitable nanocarrier for enhancing the skin permeability of RB to fight against cutaneous melanoma lesions.

In another investigation, a novel topical transferosome-oligopeptide gel formulation containing PTX and cell-penetrating peptide (CPP) (R8H3) was developed by Jiang and colleagues to treat cutaneous melanoma [338]. In this study, the authors first encapsulated the PTX in CPP-modified transferosomes, which was further incorporated into a unique oligopeptide hydrogel that acted as a reservoir, providing a prolonged skin retention time compared to PTX-CPP@transferosomal solution. The developed formulation efficiently extrudes through the channels of the stratum corneum into the epidermal layer where the melanoma cells are located. Finally, results from both in vitro cell line study and in vivo animal study revealed the PTX-CPP@transferosomal gel significantly inhibited the tumor growth compared to PTX-CPP@transferosomal solution and free PTX. Approximately 3 years later, another study on topical transferosome-oligopeptide gel formulation containing DTX was reported by the same research team to overcome the post-surgical melanoma tumor recurrence [339]. Corresponding to their previous study, the authors loaded DTX into CPP-modified transferosomes followed by incorporating it into oligopeptide hydrogel. Unlike previous study, here the authors have studied the final hydrogel for both paintability and syringeability. Finally, the DTX-CPP@tranferosomal gel displayed maximum tumor growth inhibition compared to DTX@tranferosomal gel and DTX-CPP@tranferosomal solution in the melanoma-bearing mice model, concluding that transferosome-oligopeptide gel formulation in combination with CPP could potentially enhance the skin delivery of PTX and DTX to treat cutaneous melanoma condition.

Honokiol/ transferosomes/ melanoma [340]**,** melittin/ lipoosmes/ melanoma [341]**,** berberine/ transniosomes/ skin cancer [342]**,** protein kinase C inhibitor, BRD4 PROTAC/ liposomes/ melanoma [343]**,** hispolon, doxorubicin/ liposomes/ melanoma [344]**,** phenylethylresorcinol/ liposomes, transferosomes, invasomes/ melanoma [345]**,** brucine/ ethosomes/ melanoma [346]**,** berberine chloride, evodiamine/ ethosomes/ melanoma [347]**,** 5-fluorouracil/ ethosomes/ melanoma [348]**,** doxorubicin, Au, TRP-2, polyinosinic:polycytidylic acid/ liposomes, chitosan, poly(lactide-co-glycolic acid)/ melanoma [349] are few more investigations of nanovesicles that endowed excellent anti-skin cancer efficacy.

The lipid-based NPs that have shown exemplary anti-skin cancer efficacy are summarized in Table 4.

**Table 4:** Latest investigations on lipid NPs-based therapeutic approaches for skin cancer

**Type**	**Chief composition**	**Therapeutic agent**	**Particle size and entrapment efficiency**	**In vitro cytotoxicity study**	**Animal model**	**Route of administration**	**Ref**
Solid lipid nanoparticles	Glyceryl monostearate/ Dimethyldioctadecyl ammonium bromide	Ascorbyl palmitate/ Paclitaxel	223–254 nm	Mice melanoma cell line (B16F10)	Melanoma-bearing female C57BL/6 mice	Intravenous	[350]
Solid lipid nanoparticles	Lecithin	5-Fluorouracil	137 ± 5.5–800 ± 53.6 nm	-	Melanoma-bearing male BALB/c mice	Topical	[351]
Solid lipid nanoparticles	Sodium behenate/ PVA9000	Temozolomide	273.15 ± 5 nm	Mice melanoma cell line (B16F10)	Melanoma-bearing female C57BL/6 J mice	Topical	[352]
Solid lipid nanoparticles	Glycerol palmitostearate	Doxorubicin	92 ± 2 nm	Mice melanoma cell line (B16F10)	Melanoma-bearing male BALB/c mice	Topical	[353]
Nanostructured lipid carriers	Beeswax/ Lavender oil/ Melaleuca oil	Bupivacaine	189.6–313.9 nm (lavender)/ 204.2–414.0 nm (melaleuca)	Mice melanoma cell line (B16F10)	-	Topical	[354]
Nanostructured lipid carriers	Lipid Sefsol®/ Geleol®	Silymarin	-	Mice melanoma cell line (B16F10)	Melanoma-bearing albino Swiss mice	Topical	[355]
Liposomes	Dipalmitoyl phosphatidyl choline/ Cholesterol/ Cyclodextrin/ Polyethyleneimine	Ovalbumin	207 ± 20 nm	-	Melanoma-bearing male C57BL/6 mice	-	[356]
Liposomes	Dipalmitoyl phosphatidyl choline/ Hydrogenated soy phosphatidylcholine/ Cholesterol	Doxorubicin	114–123 nm	-	Melanoma-bearing female C57BL/6 mice	Intravenous/ Intratumoral	[357]
Liposomes	Hydrogenated soy phosphatidylcholine/ Cholesterol	Epacadostat	128.1 ± 1.1 nm	Mice melanoma cell line (B16F10)	Melanoma-bearing female C57BL/6 mice	Intravenous	[358]
Liposomes	Soybean phospholipids/ Cholesterol/ Didodecyl dimethylammonium bromide/ Tocopherol PEG 1000 succinate	MicroRNAs	134 ± 1.42 nm	Human epidermoid carcinoma cell line (A431)	Squamous cell carcinoma-bearing BALB/c nude mice	Intravenous	[359]
Niosomes	Span 60/ Tween 60/ Cholesterol	Ozonated olive oil	125.34 ± 13.29 nm	Human melanoma cell line (A375)	-	Topical	[360]
Niosomes	Span 85/ Cholesterol	Ethanol extract of propolis	232 nm	Human melanoma cell line (SK-MEL)	-	Topical	[361]
Ethosomes	Ethanol/ Phospholipon 90 G/ Cholesterol	Vismodegib	559.77–562.90 nm	-	Basal cell carcinoma-bearing male Swiss albino mice	Topical	[362]
Ethosomes	Ethanol/ Soya lecithin/ Cholesterol	Silver nanoparticles/ Silk sericin	261.3 ± 5.21	Human epidermoid carcinoma cell line (A431)	Squamous cell carcinoma-bearing BALB/c mice	Topical	[363]

## Drug delivery patch for skin cancer therapy

Recently, drug-delivery patches have grasped the interest of many scientists due to their ability to not only deliver the therapeutic agents to the systemic circulation but also to the local skin region for prolonged periods to treat diverse skin conditions, including cutaneous cancer [364]. Interestingly, these drug-delivery topical/transdermal patches can be loaded with either free anticancer drugs or NPs imbibed anticancer drugs for efficient skin cancer therapy. A recent study by Song and colleagues developed tumor antigens-loaded ethosomes and further imbibed them in a polyvinyl alcohol (PVA) and silk fibroin (SF) based nanofibrous patch for treating melanoma [365]. In this study, the authors have modified the surface of ethosomes with mannosylated polyethyleneimine to target dendritic cells. The results revealed that the developed nanofibrous patch sufficiently inhibited the tumor growth in melanoma-bearing mice model. Further, the combination of vaccine patch and anti-PD-1 exhibited synergistic anti-melanoma activity, encouraging the combinatorial delivery of vaccine and anti-PD-1 for efficient skin cancer therapy. Another study by the same team prepared mRNA vaccines and anti-PDL1 siRNA-loaded ethosomes and then incorporated them into SF-based electro-spun transdermal patch for melanoma therapy [366]. Unlike their previous study, the authors have modified the surface of ethosomes with mannosylated chitosan to target dendritic cells. The results from animal studies showed that the developed nanofibrous patch could efficiently and non-invasively treat melanoma conditions. A recent investigation by Guadagno and teammates developed an Au complex-loaded PCL-based electro-spun nanofibrous topical patch for the treatment of melanoma [367]. The results revealed that the developed nanofibers exhibited significant cell death within 48 h, encouraging the potential of a metal-based topical patch in melanoma therapy. Recently, studies have demonstrated the synergistic potential of metallic NPs and phytoconstituents to treat skin cancer. In this context, a study by Ekambaram and co-workers synthesized green-based titanium dioxide nanorods (TiO_2_ NRs) and further incorporated them into PVA-based nanofibrous patch along with resveratrol (TR@NFs) to treat non-melanoma skin cancer [368]. The in vitro cytotoxicity study in A431 cell lines exhibited decreased IC_50_ value for developed TR@NFs compared to free resveratrol. At 500 µg/ml concentration, TR@NFs showed reduced cell viability in A431 cell lines than T@NFs and plain NFs, exploiting the synergistic effect of TiO_2_ NRs and resveratrol in non-melanoma skin cancer.

Although topical/transdermal patches can efficiently treat skin cancer conditions, their preparation technique, loading dose, and non-customizable size of the patch are the most significant drawbacks that need to be potentially addressed. In this contemplate, a recent study by Shao et al. investigated a personalized 3D printable topical patch through the guidance of dermoscopy for treating diverse skin conditions [369]. Interestingly, both hydrophilic and lipophilic drugs can be precisely printed on the patterned patch with the help of an inkjet printer according to patients’ conditions, i.e., size and location of the lesion. With this strategy, the limitations associated with conventional patch fabrication can be potentially resolved, which indirectly paves the road for efficient skin cancer therapy.

## Microneedle patch for skin cancer therapy

Microneedles (MNs) are advanced drug delivery patches whose needle height ranges from 100 to 2000 µm [370]. They are considered third-gen topical/transdermal drug delivery systems due to their profound ability to overcome the drawbacks of many topical/transdermal formulations such as gel, cream, lotion, ointment, conventional patch, spray, etc. [253]. The MN patches are capable enough to physically disrupt the skin’s most rigid barrier, “Stratum corneum”, to deliver a wide range of anticancer drugs or NPs imbibed therapeutic agents directly into the dermal layer to treat cutaneous skin cancer. However, when there is a skin cancer metastasis, these MNs can be utilized to deliver a wide range of drug-loaded NPs into systemic circulation as a substitute for invasive techniques (intravenous, subcutaneous, or intramuscular injections) [371–373]. Previously, Demartis and colleagues developed Rose Bengal (RB) loaded transferosomes (T) for treating cutaneous melanoma by overcoming their permeability issue [337]. However, the same team further extended their work by loading RB@T into PVA-polyvinylpyrrolidone (PVP) based dissolving MNs to enhance the residence of RB in the cutaneous melanoma region compared to plain transferosomal formulation [374]. The developed RB@T was 62 nm in size with a zeta potential value of -38.5 mV. Further, the RB loading in transferosomal formulation was estimated to be 110%. Thereafter, the RB@T@MNs patch consisted of 600 pyramidal needles with an individual needle height of approx. 750 µm. The results from drug content determination studies revealed that the free RB@MN patch contained 139 ± 22 µg of RB, whereas RB@T@MN patch loaded just 64 ± 8 µg of RB. The developed MNs exhibited good mechanical strength with not more than a 10% reduction in needle height upon application of 32 N force. Coming to the insertion studies, the developed MNs reached the maximum depth of 381 µm in a Parafilm skin simulant model. The results from the insertion study of MNs in the ex-vivo model exhibited a penetration depth of 400–450 µm. Finally, the RB@T@MNs showed complete dissolution of needles within 5 min in an ex vivo set-up, whereas RB@MNs liquified after 10 min. The fastest dissolution rate of RB@T@MNs could be due to the presence of surfactant (Span 80) in transferosomal formulation. Nevertheless, the dermatokinetic study exhibited that the developed RB@T@MNs could be more competent in treating melanoma conditions than RB@MNs and RB@T. Considering all these results, combining nano- and micro-based drug delivery systems could be the most predominant approach to enhance the therapeutic efficacy of anticancer agents in cutaneous melanoma therapy [374].

BRAF is one of the most commonly mutated genes in melanoma. Therefore, researchers have used BRAF siRNA (siBraf) as a frontline treatment approach [375]. However, due to the hydrophilicity and large molecular weight (13 kDa), siBraf is facing skin permeability issues in reaching the melanoma site. To overcome this drawback, Ruan and teammates, for the first time, developed the siBraf-octaarginine (R8) (cell-penetrating peptide) based NPs (R8-siBraf) and further coated them on stainless steel MNs (R8-siBraf@MNs) for the efficient treatment of cutaneous melanoma [376]. The size of developed R8-siBraf NPs was found to be 353 nm. The coated MNs consisted of 10 × 10 conical arrays with individual needle heights of approx. 750 µm. The in vivo study revealed that the developed R8-siBraf@MNs released 90% of siRNA into the skin within 5 min of insertion. Further, the in vivo insertion depth of MNs was found to be approx. 300 µm using Rhodamine B coated MNs. The results from in vitro cell line studies using A375 cells exhibited that R8-siBraf NPs can enhance the BRAF gene silencing, thereby reducing the cell viability compared to polyethyleneimine-siBraf NPs. Finally, the developed R8-siBraf@MNs significantly reduced the tumor size in melanoma-bearing mice models via inducing apoptosis and inhibiting A375 cell proliferation, making them the most appropriate candidates for melanoma therapy.

**Fig. 10:**
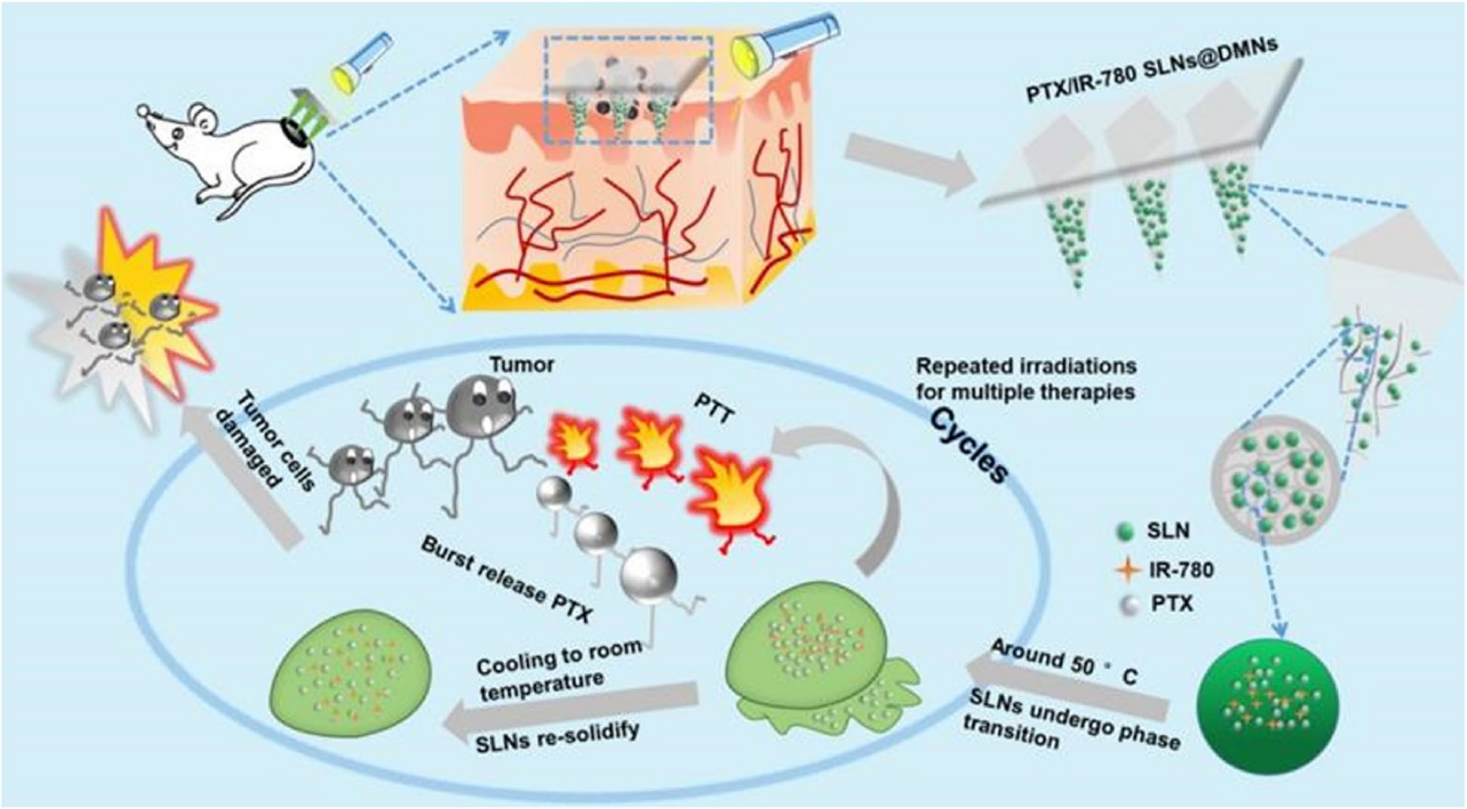
Diagrammatic representation of spatiotemporally controlled pulsatile release microneedle drug delivery system for the treatment of melanoma, reproduced with permission from [377], licensed under CC BY 4.0

Recently, Qin and co-workers developed nano- and micro-based delivery systems to achieve chemo-photothermal therapy in skin melanoma [377]. In this study, the authors first developed the PTX (chemo) and IR-780 (PTT agent) loaded thermo-responsive SLNs (PI@SLNs) and further incorporated them into a dissolving MN system to develop a unique spatiotemporally controlled delivery system that can furnish repeated controlled drug delivery for long-term melanoma therapy. Upon application of PI@SLNs@MNs into the tumor site, PI@SLNs were released and accumulated in the melanoma site. Further, the irradiation of NIR light triggers the IR-780 to convert the light energy into heat, resulting in in-situ phase transformation of SLNs, leading to PTX release. However, under no NIR irradiation, the reduced temperature is witnessed, facilitating the re-solidification of SLNs, further inhibiting PTX delivery (Fig. 10). With this system, multiple doses have been achieved in a single administration, which exhibited significant tumor inhibition (100% tumor eradication in 30 days) in an in vivo model compared to intratumoral and intravenous administration of PTX/IR-780 SLNs. These results revealed that nano- and micro-based chemo-photothermal therapy is a unique way to treat melanoma with relatively less toxicity [377].

Hypericin (Hy) is a natural photosensitizing agent (PDT) that has received significant attention due to its ability to produce high-efficiency superoxide anions and singlet oxygen species upon light irradiation [378]. However, their therapeutic efficacy has been substantially hindered due to the hydrophobicity and poor skin permeability. To overcome this issue, Abd-El-Azim and colleagues developed Hy-loaded lipid nanocapsules (Hy@LNs) and further delivered them to the non-melanoma skin cancer site through hollow MN (HMN) to improve the efficacy of localized PDT [379]. The particle size of developed Hy@LNs was 47 nm with negative zeta potential. The HMN system consisted of a single needle with a height of 1300 µm. The optical coherence tomography (OCT) analysis revealed that the developed HMN penetrated through a depth of 1045 µm, which is approx. 95% of total needle height. Upon delivery through HMNs, these Hy@LNs enhanced tumor cell uptake, furnishing improved PDT therapy in non-melanoma skin cancer conditions. Finally, the developed Hy@LNs delivered via HMN system exhibited remarkable inhibition of tumor growth upon light irradiation (595 nm) in a nude mice model. Overall, based on these results, it is witnessed that NPs delivered through MNs could furnish remarkable cutaneous skin cancer therapy than free drug-loaded MNs, free drug-loaded conventional topical/transdermal formulations, and NPs-based conventional topical/transdermal formulations. Nevertheless, the NPs imbibed MNs could deliver the drugs into the systemic circulation to treat metastasized skin cancer by potentially overcoming the drawbacks associated with hypodermic needle-based injections, such as pain and inconvenience [379].

Some of the recent MNs-based approaches that demonstrated efficient skin cancer therapy include 5-fluorouracil, indocyanine green/ PCL NPs/ HA MNs/ epidermoid carcinoma, melanoma [280], Cu-doped polydopamine NPs (PDT)/ PVP-PVA MNs/ melanoma [380], STAT3 siRNA/ dextran-PVP-HA MNs/ melanoma [381], curcumin, indocyanine green/ HA-alginate-gelatin MNs/ melanoma [382], doxorubicin, trametinib/ dextran methacrylate hydrogel MNs/ melanoma [383].

## Patents and clinical trials

There are many journal publications reporting on innovative nano-based therapeutic approaches for the treatment of skin cancer. However, few researchers have protected their inventions/innovations through patents with the intention of taking those nano-based therapeutic approaches to clinical trials followed by commercialization. Some recently published patents to treat skin cancer via nanotherapeutics are illustrated in Table 5 while excluding those patents that made general claims for treating all types of cancer conditions without performing in vitro or in vivo studies on skin cancer models. Out of hundreds of patents, few also entered clinical trials, as illustrated in Table 6. Further, it is clearly observable that most of the clinical trials have been performed for already marketed nano-based therapeutics (used for other conditions like breast cancer, pancreatic cancer, etc.) alongside different anticancer agents (immunotherapeutic, targeted therapeutic, or chemotherapeutic agents) to explore the combinatorial/ synergistic effect on skin cancer.

**Table 5:** Patents published on NPs-based therapeutic approaches for skin cancer

**Type of nanoparticle/ Therapeutic agent**	**Patent no**	**Inventors and Applicants**	**Title of the invention**	**Type of study**	**Year of publication**	**Ref**
Organometallic complex (Iron-based)	US2010119608A1	Fred et al	Synthesis of pH-sensitive, acid-stable metal-binding nanoparticles	In vivo	2010	[384]
Cerium oxide nanoparticle	US2013337070A1	Peter et al./ Peter et al	Coated nanoparticle therapy for skin cancer	In vivo	2013	[385]
Silver-silverbromide-titanium dioxide nanoparticle	KR20130057744A	Woo et al./ Univ Soongsil Res Consortium	Composition for treating cancer containing Ag/AgBr/TiO_2_ nanoparticles that be activated under visible light	In vivo	2013	[386]
Polymeric nanoparticle (Albumin)/ Bevacizumab	CA2917407A1	Svetomir And Wendy/ Mayo Foundation	Complexes containing albumin-containing nanoparticles and antibodies to treat cancer	In vivo	2014	[387]
Polymeric micelle/ Doxorubicin, Cisplatin	CN104784700A	Huayu et al. Changchun Applied Chemistry	Medicine co-carried compound, micelle and preparation method of micelle	In vitro	2015	[388]
Niosome/ Doxorubicin	RU2600164C2	Aleksandrovich	Doxorubicin and organosilicon nanoparticles-niosomes-based pharmaceutical gel for skin cancer treating	-	2016	[389]
Carbon nanotube	WO2018008825A1	Oh et al./ Korea Advanced Inst Sci & Tech	X-ray brachytherapy system for keloid and skin cancer treatment using carbon nanotube-based X-ray tube	-	2018	[390]
Gold nanoparticle	US2019142980A1	Angela et al./ Ricerche et al	Cells loaded with gold nanoparticles for use in the diagnosis and/or treatment of melanoma	In vivo	2019	[391]
Silver prussian blue nanoparticle	US10231996B2	Sudip and Ranjan/ Council Scient Ind Res	Biocampatible polymer coated silver prussian blue nanoparticles (SPB-NPs: Ag3[Fe(CN) 6])	In vivo	2019	[392]
Polymeric micelle	CN110339368A	Zhiyuan et al./ Univ Suzhou	Preparation method of reduction-response targeting polyethylene glycol-polycarbonate maytansine prodrug micelle	In vivo	2019	[393]
Polymeric nanoparticle (Albumin)/ Paclitaxel, Trastuzumab	US10406224B2	Svetomir And Wendy/ Mayo Found Medical Education & Res	Nanoparticle complexes of paclitaxel, trastuzumab, and albumin	In vivo	2019	[394]
Carbonyl iron-sulfur cluster nanoparticle	CN111281858A	Hong et al./ Univ Shanghai	Application of carbonyl iron-sulfur cluster compound nanoparticles in drug preparation	In vivo	2020	[395]
Polymeric nanoparticle (PLGA)/ Apatinib	CN111150718A	Ju et al./ Univ Qingdao	PLGA/poloxamer nanoparticles entrapping apatinib, production method and application	In vivo	2020	[396]
Polymeric nanoparticle (Albumin)/ Paclitaxel, Bevacizumab	US10765741B2	Svetomir And Wendy/ Mayo Found Medical Education & Res	Methods for treating VEGF-expressing cancer using preformed nanoparticle complexes comprising albumin-bound paclitaxel and bevacizumab	In vivo	2020	[397]
Polymeric nanoparticle (DSPE-PEG2000)/ Temozolomide	CN111481526A	Guan et al./ Univ Xuzhou Medical	Cell-penetrating peptide modified drug-loaded thermosensitive nanoparticle and application thereof in resisting melanoma	In vitro	2020	[398]
Liposome/ Catalase, anti-PDL1	CN110974957A	Shichen et al./ Univ Beijing and Beijing Hongxin Stem Cell Biotechnology Co Ltd	Application of catalase-entrapped liposome connected with PD-L1 antibody in preparation of tumor treatment drugs	In vivo	2020	[399]
Polymeric micelle/ Acetogenins	ES2826205A1	Teresa et al./ Univ Cadiz	Procedure for obtaining a pharmaceutical composition using acetogenins with supramolecular polymeric micelles for the treatment of skin cancer	In vitro	2021	[400]
Liposome/ Dacarbazine, Veirofenib (Vemurafenib)	CN113244174A	Qianqian et al./ Guangdong Laboratory of Southern Ocean Science and Eng Zhanjiang	Melanoma chemotherapy drug-loaded nano-liposome and preparation method thereof	In vitro	2021	[401]
Liposome/ Doxorubicin, Trabectedin	WO2022115075A1	Güliz and Şenay/ Ege Ueniversitesi	Targeted nanoparticles carrying dual drugs in the treatment of melanoma	In vivo	2022	[402]
Gold nanoparticle/ Palladium nanoparticle/ Platinum nanoparticle/ Bimetallic gold–palladium nanoparticle/ Bimetallic gold-platinum nanoparticle	US2022218741A1	Medina et al./ Univ Northeastern	Cell-mediated synthesis of noble metal oxide nanoparticles and biomedical applications thereof	In vitro	2022	[403]
Polymeric nanoparticle (DSPE-PEG)/ Photothermal agent	WO2022134862A1	Lei et al./ Univ South China Tech	Organic conjugated polymer photo-thermal reagent for treating malignant melanoma, nanoparticle, and preparation method therefor and use thereof	In vitro	2022	[404]

**Table 6:** Clinical trials conducted for the management of skin cancer via nanotherapeutics (Source: ClinicalTrials.gov)

**Type of nanoparticle/ Therapeutic agent**	**Trial no**	**Title of the study**	**Condition**	**Phase**	**Country**	**Status**	**Start–End year**
Liposome/ Interferon alfa-2b, Melanoma vaccine	NCT00004104	Vaccine therapy plus interleukin-2 with or without interferon alfa-2b in treating patients with stage iii melanoma	Melanoma	Phase 2	United States	Completed	1998–2000
Albumin-nanoparticle/ Paclitaxel (ABI-007)	NCT00081042	ABI-007 in treating patients with inoperable locally recurrent or metastatic melanoma	Melanoma	Phase 2	United States	Completed	2004–2010
Liposome/ Vincristine	NCT00145041	Pharmacokinetic study of liposomal vincristine in patients with malignant melanoma & hepatic dysfunction	Melanoma	Phase 1	United States	Completed	2005–2007
Albumin nanoparticle/ Paclitaxel (ABI-007)	NCT00404235	Carboplatin and ABI-007 in treating patients with stage iv melanoma that cannot be removed by surgery	Melanoma	Phase 2	United States	Completed	2006–2010
Albumin nanoparticle/ Paclitaxel	NCT00626405	Bevacizumab and temozolomide or bevacizumab and paclitaxel albumin-stabilized nanoparticle formulation and carboplatin in treating patients with stage iv malignant melanoma that cannot be removed by surgery	Melanoma	Phase 2	United States	Completed	2008–2012
Nanoparticle/ Docetaxel (BIND-014)	NCT01300533	A study of BIND-014 given to patients with advanced or metastatic cancer	Skin cancer	Phase 1	United States	Completed	2011–2016
Albumin nanoparticle/ Paclitaxel (Abraxane®)	NCT02158520	nab-Paclitaxel and bevacizumab or ipilimumab as first-line therapy in treating patients with stage iv melanoma that cannot be removed by surgery	Melanoma	Phase 2	United States	Completed	2013–2019
Albumin nanoparticle/ Paclitaxel (Abraxane®)	NCT02020707	nab-Paclitaxel and bevacizumab in treating patients with unresectable stage iv melanoma or gynecological cancers	Melanoma	Phase 1	United States	Recruiting	2014-present
Albumin nanoparticle/ Paclitaxel (Abraxane®)	NCT02495896	Recombinant EphB4-HSA fusion protein with standard chemotherapy regimens in treating patients with advanced or metastatic solid tumors	Head and neck squamous cell carcinoma	Phase 1	United States	Active, not recruiting	2015-present
Lipid nanoparticle/ mRNA-2752	NCT03739931	Dose escalation study of mRNA-2752 for intratumoral injection to participants in advanced malignancies	Melanoma	Phase 1	United States, Israel	Recruiting	2018-presnt
Topical nanoparticle ointment/ Paclitaxel (SOR007)	NCT03101358	Study of topical SOR007 ointment for cutaneous metastases	Cutaneous metastases from non-melanoma cancer	Phase 1 & 2	United States	Completed	2018–2020
Quantum dots	NCT04138342	Topical fluorescent nanoparticles conjugated somatostatin analog for suppression and bioimaging breast cancer	Skin cancer	Phase 1	Egypt, Saudi Arabia	Recruiting	2019-present
Hafnium oxide-containing nanoparticle (NBTXR3)	NCT04834349	Re-irradiation with NBTXR3 in combination with pembrolizumab for the treatment of inoperable locoregional recurrent head and neck squamous cell cancer	Head and neck squamous cell cancer	Phase 2	United States	Active, not recruiting	2021-present
Hafnium oxide-containing nanoparticle (NBTXR3)	NCT04862455	NBTXR3, radiation therapy, and pembrolizumab for the treatment of recurrent or metastatic head and neck squamous cell cancer	Head and neck squamous cell carcinoma	Phase 2	United States	Recruiting	2021-presnt
Gadolinium-based nanoparticle	NCT04899908	Stereotactic brain-directed radiation with or without AGuIX gadolinium-based nanoparticles in brain metastases	Metastasized melanoma	Phase 2	United States	Recruiting	2021-presnt
Liposome/ mRNA vaccine	NCT05264974	Novel RNA-nanoparticle vaccine for the treatment of early melanoma recurrence following adjuvant anti-PD-1 antibody therapy	Melanoma	Phase 1	United States	Not yet recruiting	2022-present
Hafnium oxide-containing nanoparticle (NBTXR3)	NCT04892173	NBTXR3 with or without cetuximab in LA-HNSCC	Head and neck squamous cell carcinoma	Phase 3	United States, Belgium, Bulgaria, France, Georgia, Spain	Recruiting	2022-presnt

## Conclusion

Nanotechnology has opened a new door in the medical field to overcome several impediments associated with conventional skin cancer treatment modalities. Due to the ability of nanoparticles to act as anticancer agents, drug carriers, tumor-targeting moiety, skin permeability enhancers, and so on, they are considered suitable candidates for efficient skin cancer therapy. As witnessed through numerous research reports, nanoparticle-based therapeutic approaches (inorganic, polymer, and lipid-based nanoparticles) have endowed significant improvement in the skin cancer therapy compared to conventional treatment approaches. The NPs have changed the outlook of immunotherapy, targeted therapy, and chemotherapy in terms of their required dose, therapeutic efficacy, toxicity, stability, and so on. Specifically, for expensive cancer treatments such as immunotherapy and targeted therapy, improving the therapeutic efficacy with as low a dose as possible is highly important in bringing down the cost of an overall treatment. Furthermore, the nanoparticles have allowed us to treat the most aggressive metastasized skin tumors via various routes of administration (intravenous, intratumoral, oral, and transdermal). However, the initial stages of skin cancer lesions can be simply treated with minimally or non-invasive routes such as topical (gel, cream, and microneedles) without much toxicity complications. Despite all these superiorities, it is unfortunate that there are still no commercialized nano-based skin cancer therapeutics. With this review, it is expected to see commercial nanotherapeutics for skin cancer therapy shortly, similar to currently existing commercial nanoformulations for other cancers.

## Data Availability

Not applicable

## Abbreviations

AAD: American Academy of Dermatology
BCC: Basal cell carcinoma
SCC: Squamous cell carcinoma
FDA: Food and Drug Administration
EMA: European Medicines Agency
HPV: Human papillomavirus
PTT: Photothermal therapy
NPs: Nanoparticles
EPR: Enhanced permeability and retention
PDT: Photodynamic therapy
MSNs: Mesoporous silica nanoparticles
IUPAC: International Union of Pure and Applied Chemistry
CMC: Critical micelle concentration
CP: Cisplatin
MTT: (3-(4, 5-Dimethylthiazolyl-2)-2, 5-diphenyltetrazolium bromide)
DTIC: Dacarbazine
RVT: Resveratrol
siRNA: Small interfering RNA
NIR: Near-infrared light
CNTs: Carbon nanotubes
MDSC: Myeloid-derived suppressor cells
CUR: Curcumin
